# GPX4 Inhibitor Resistance and Metastatic Features in Triple‐Negative Breast Cancer

**DOI:** 10.1002/advs.202523198

**Published:** 2026-02-17

**Authors:** Marie Sabatier, Mayher Kaur, Milena Chaufan, Felix‐Levin Hormann, Luiza Martins Nascentes Melo, Mario Palma, Jordan Torpey, Yanshan Liang, Alanis Carmona, Cameron Fraser, Midori Flores, Krystina J. Szylo, Mahsa Yavari, Sheng Hui, Alpaslan Tasdogan, Sven Heiles, Jessalyn M. Ubellacker

**Affiliations:** ^1^ Department of Molecular Metabolism Harvard T.H. Chan School of Public Health Boston Massachusetts USA; ^2^ Leibniz‐Institut Für Analytische Wissenschaften ‐ ISAS ‐ E.V. Dortmund Germany; ^3^ Lipidomics Faculty of Chemistry University of Duisburg‐Essen Essen Germany; ^4^ Department of Dermatology University Hospital Essen & German Cancer Consortium (DKTK) Essen Germany; ^5^ Ludwig Center at Harvard Boston Massachusetts USA

**Keywords:** ferroptosis, lipid peroxidation, metastases, tumor microenvironment

## Abstract

Leveraging ferroptosis as a cancer therapy has faced challenges due to the limited bioavailability and systemic toxicities of small‐molecule ferroptosis modulators. Small molecule inhibitors such as RSL3 and ML210 trigger ferroptosis by targeting glutathione peroxidase 4 (GPX4), a key enzyme that neutralizes lipid peroxides. While many studies have focused on targeting primary tumors, much less is known about the extent to which GPX4‐inhibitor resistance may contribute to metastasis. To address this, we cultured triple‐negative breast cancer cell lines with GPX4 inhibitors to generate cell lines (M231, 4T1) that were resistant to GPX4 inhibitors (GPX4i). Tumors derived from GPX4i‐resistant cells compared to parental cells had unique metabolic and lipidomic profiles, were associated with a shift toward an epithelial‐like state (decreased vimentin, increased EpCAM expression), formed decreased spontaneous metastases from primary tumors, but had no differences in overall metastatic burden upon intravenous injection. Collectively, these data demonstrate that long‐term maintenance with GPX4‐inhibitors in vitro leads to altered metastatic profiles in vivo.

## Graphical abstract

This study establishes breast cancer cell lines that are resistant to glutathione peroxidase 4 inhibitors (GPX4i) in vitro and investigates the metastatic capacity of the GPX4i‐resistant versus parental lines in vivo. GPX4i‐resistant cells survive upon intravenous injection, but lose capacity to spontaneously metastasize. In vivo characterization of tumors derived from GPX4i‐resistant lines reveals heterogenous metabolic and lipid adaptations across models.

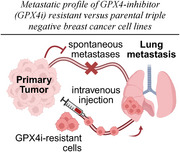

## 1 Introduction

Ferroptosis, a regulated form of non‐apoptotic cell death, is currently being explored in the context of cancer therapy but is associated with significant challenges [1, 2, 3]. These include off‐target effects and toxicity to normal cells caused by small‐molecule ferroptosis inducers, as well as a lack of in vivo pre‐clinical studies to guide effective application, among other obstacles [4]. Despite these limitations, certain cancer subtypes appear particularly susceptible to ferroptosis in vitro, suggesting the possibility for selectively targeting specific cancers.

Triple‐negative breast cancer (TNBC), a highly aggressive subtype that lacks estrogen receptor (ER), progesterone receptor (PR), and human epidermal growth factor receptor 2 (HER2) expression, has limited treatment options and is associated with early metastasis and poor prognosis. Compared to other breast cancer subtypes, TNBC has increased sensitivity to ferroptosis induction in vitro and in primary tumors [5, 6, 7]. However, the role of ferroptosis in TNBC metastatic capacity remains largely unexplored.

Ferroptosis is characterized by the rupture of cellular membranes due to the accumulation of lipid peroxides in membrane phospholipids [8, 9]. The enzyme glutathione peroxidase 4 (GPX4) is a key antioxidant defense against ferroptosis and uses glutathione (GSH) as a co‐factor to reduce lipid peroxides into non‐toxic lipid alcohols [10, 11]. GPX4 is essential for the survival of most cancer cells in vitro, and its inhibition with small molecules ((1S,3R)‐RSL3 (RSL3), ML210, JKE‐1674, among others) triggers ferroptosis [12, 13]. RSL3 irreversibly inhibits GPX4 by covalently modifying its selenocysteine residue through an electrophilic interaction [12]. However, this same mechanism of RSL3 can impact other selenoproteins and lacks metabolic stability, making it unsuitable for in vivo systemic administration [14, 15, 16, 17]. ML210, as a nitrile‐oxide electrophile that inhibits GPX4, has increased selectivity and is being more broadly used in the field of ferroptosis but also has limited systemic efficacy in vivo [18]. Because much of the foundational work in the ferroptosis field has relied on RSL3, we focus primarily on RSL3 in this study while using ML210 to evaluate the extent to which our observed effects reflect GPX4 inhibition rather than RSL3‐specific off‐target effects.

Metastasis of cancer cells from the primary tumor to distant organs is a complex, multi‐step cascade involving invasion, intravasation, circulation, extravasation, and colonization [19]. Each of these steps exposes cancer cells to distinct metabolic and microenvironmental conditions, including shifts in redox balance, that differ markedly from those in the primary tumor microenvironment [20, 21]. Although targeting GPX4 has been shown to inhibit tumor growth in certain primary tumors [22, 23], recent studies suggest that circulating melanoma cells, but not melanoma cells from primary tumors or lymph nodes, are dependent on GPX4 for survival [24]. This raises the possibility that ferroptosis induction may be particularly effective at specific steps of the metastatic cascade.

Because systemically administered GPX4 inhibitors would inevitably affect both primary and disseminating cancer cells, it is critical to understand the impact of targeting GPX4 at each step of the metastatic cascade. Yet, most of what we know about ferroptosis comes from in vitro studies using small‐molecule GPX4 inhibitors such as RSL3 and ML210—compounds that, despite their limitations for in vivo use, have formed the foundation for mechanistic work in ferroptosis. As the field now shifts toward investigations with more bioavailable GPX4 inhibitors (e.g., Compounds 18 and 28 [25, 26, 27, 28], among others), there is a pressing need to translate the foundational *in* vitro insights from RSL3 and ML210 to the in vivo contexts of cancer metastasis, where cancer cells face complex physiological pressures not captured in cell culture.

In this study, we used RSL3 and ML210 as tool compounds to investigate how chronic exposure and resistance to these agents in vitro affected metastatic behavior in vivo, aiming to connect the extensive in vitro ferroptosis literature with in vivo metastasis profiles. Specifically, we generated GPX4‐inhibitor (GPX4i) resistant TNBC cells and evaluated their metastatic behavior at distinct steps of the metastatic cascade. This strategy allows us to assess the role of ferroptosis in metastasis and provides a functional readout that reflects how sensitivity or resistance to ferroptosis influences metastatic capacity in vivo during TNBC growth and dissemination to distant organs.

## 2 Results

### 2.1 Generation of TNBC Cells Resistant to GPX4 Inhibition

To investigate the extent to which resistance to GPX4 inhibitors in vitro alters metastatic features of TNBCs in vivo, we first generated GPX4‐inhibitor (GPX4i) resistant 4T1 (murine TNBC) and MDA‐MB‐231 (M231, human TNBC) cells. To do this, 4T1 or M231 cell lines were exposed in vitro to continuous treatments with vehicle, RSL3, or ML210. Cells were exposed to increasing concentrations of RSL3 (from 50 nm to 1 µm) or ML210 (from 500 nm to 30 µm) over a three‐month period. These concentrations are within the optimal range to induce ferroptosis while limiting non‐specific cell toxicity [37], and are still rescuable by ferroptosis inhibitors. Despite the longer passage time, the immortalized 4T1 and M231 cell lines did not exhibit senescence‐associated changes and maintained similar proliferation rates in later passages.

**FIGURE 1:**
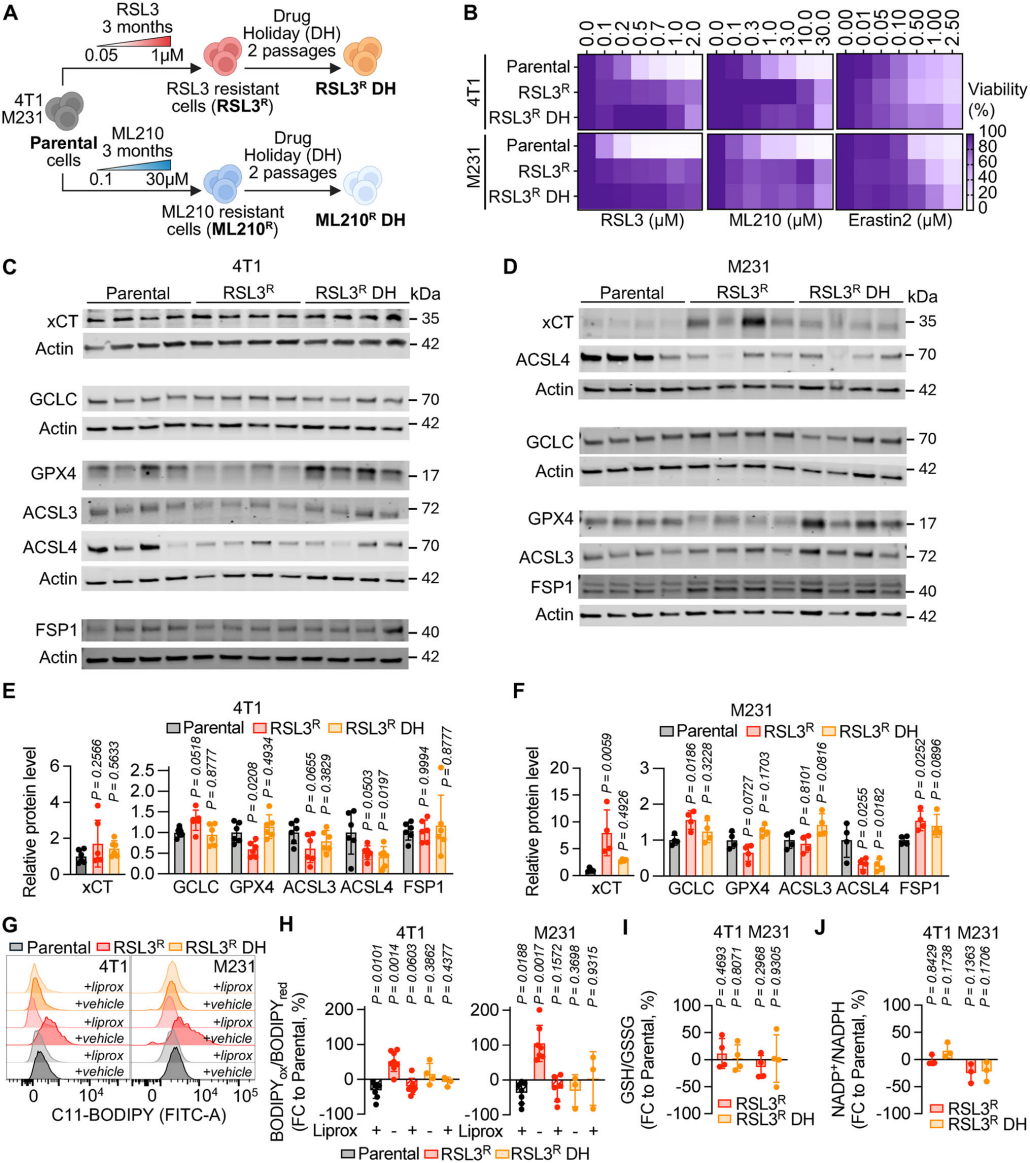
Generation and in vitro characterization of GPX4‐inhibitor‐resistant TNBC cells. (A) Experimental scheme of the generation of 4T1 and M231 cell lines resistant to small molecule GPX4 inhibitors (*n* = 3 independent generations) (B) Parental, RSL3^R^, and RSL3^R^ DH cell lines were incubated with indicated doses of RSL3, ML210, and Erastin2 for 48 h. Cell viability was assessed using an absorbance‐based assay (MTT), and results are expressed as a ratio normalized to the untreated condition (*n* = 3–8 independent experiments). (C–F) Immunoblot analyses of protein extracts from 4T1 (C) and M231 (D) parental, RSL3^R^, and RSL3^R^ DH collected after 24 h. Protein level quantifications relative to the parental cells are represented in (E,F) (*n* = 4–6 independent protein extracts). (G,H) Parental, RSL3^R,^ and RSL3^R^ DH cell lines were incubated with 1 µm Liprox or vehicle for 24 h. Lipid peroxidation was evaluated by C11‐BODIPY 581/591 staining. Representative plots (G) and BODIPYox/BODIPYred, ratio of oxidized to reduced BODIPY relative to parental control group (H) are shown (*n* = 4–8 independent experiments). (I) GSH/GSSG ratio in 4T1 and M231 RSL3^R^ and RSL3^R^ DH relative to the parental control group collected after 24‐hours (*n* = 4 independent experiments). (J) NADP^+^/NADPH ratio in 4T1 and M231 RSL3^R^ and RSL3^R^ DH relative to the parental control group collected after 24‐hours (*n* = 3 independent experiments). Data in (B) are displayed as mean. Data in (E,F) are displayed as mean ± s.d. and were analyzed by one‐way ANOVA and Dunnett's multiple comparison. Data in (H,I,J) are displayed as mean ± s.d. and were analyzed by one‐sample *t*‐test.

GPX4i‐resistant cells that survived this selective pressure were expanded and maintained in culture with rsl3 (RSL3^R^) or ML210 (ML210^R^) or were subjected to a drug holiday (DH) during which cells were not exposed to the drug for two passages (i.e., 4 days total, RSL3^R^ DH and ML210^R^ DH) (Figure 1A). GPX4i‐resistant cells compared to their parental counterparts had increased viability upon GPX4 inhibitor treatment, reflected by an increase in the IC50 values of RSL3 or ML210 in the GPX4i‐resistant lines (Figure 1B; Figure S1A–D). GPX4‐inhibitor‐resistant cells displayed significantly higher viability upon reciprocal treatment with either ML210 applied to the RSL3^R^ cells or RSL3 applied to the ML210^R^ cells, thus confirming broader GPX4 resistance beyond specific resistance to RSL3 or ML210 alone (Figure 1B; Figure S1A,E–G).

### 2.2 In Vitro Characterization of Ferroptosis Regulators in GPX4‐Inhibitor Resistant Lines

We characterized the expression levels of the key ferroptosis regulators in these paired lines including GPX4 and ferroptosis suppressor protein 1 (FSP1). FSP1 suppression of ferroptosis is mediated by ubiquinone as FSP1 catalyzes the regeneration of CoQ_10_, which traps lipid peroxyl radicals that mediate lipid oxidation [29, 30, 31, 32, 33, 34]. FSP1 has recently been shown to significant relevant in the metastatic context, where microenvironmental shifts can increase FSP1‐dependencies [35, 36]. We also characterized expression levels of the cystine/glutamate antiporter xCT, glutamate‐cysteine ligase (GCLC), and acyl‐CoA synthetase long‐chain family members 3/4 (ACSL3/ACSL4), which are responsible for MUFA and PUFA incorporation into membrane PLs, respectively [32, 38]. RSL3^R^ cells from both the 4T1 and the M231 lines had reduced GPX4 protein levels compared to the parental lines. These changes were transient and were not observed in the RSL3^R^ cells that were no longer maintained under selection pressure (RSL3^R^ DH) (Figure 1C–F; Figure S4A). Consistently, the same transient reduction of GPX4 protein levels was observed in ML210‐resistant lines (Figure S4B,C).

We observed a reduction in ACSL4 in both the RSL3^R^‐resistant and DH cells (Figure 1C–F; Figure S4A). However, ACSL3 was either similar or slightly, but non‐significantly, increased in RSL3^R^ DH, but not ML210^R^ DH lines, compared to parental cells from 4T1 or M231 (Figure 1C–F; Figure S4A–C). FSP1, xCT, and GCLC protein levels were increased only in RSL3^R^ cells derived from the M231 cell line (Figure 1C–F; Figure S4A–C). Taken together, findings that were consistent in both the 4T1 and M231 lines demonstrate that the GPX4i‐resistant cells transiently have decreased GPX4 expression that returns to similar levels as observed in the parental lines upon removal of RSL3 or ML210. The readily reversible adaptations of the GPX4i‐resistant lines suggest the resistant phenotype is likely not driven by permanent genetic alterations or selection.

GPX4i‐resistant MDA‐MB‐231 cells, but not 4T1 cells, were more resistant to Erastin2, a ferroptosis inducer that inhibits system xCT, with this effect being most pronounced following a drug holiday (Figure 1B; Figure S1H–J). Although the M231 RSL3^R^ lines had increased xCT expression whereas the 4T1 RSL3^R^ lines did not, which could potentially account for their Erastin2 resistance, the RSL3^R^ DH lines remained resistant to Erastin2 despite having xCT expression levels comparable to those of the parental cells (Figure 1E,F).

To better understand what might be driving the resistance to ferroptosis‐inducing agents in the GPX4i‐resistant lines, we next conducted comprehensive in vitro viability assays to characterize cell survival differences in the parental, RSL3^R^, or RSL3^R^ DH M231 or 4T1 lines with combinations of ferroptosis‐inducing agents. These agents included ML210, Erastin, BSO (which inhibits GCLC, the rate‐limiting enzyme for de novo glutathione synthesis) administered either alone or in combination with the FSP1 inhibitors viFSP1 (4T1 and M231 lines) or FSEN1 (M231 line only, given the human specificity of this agent). Although FSP1 has shown single‐agent targetability in vivo [35, 36], effective inhibition in vitro typically requires loss of GPX4 activity, providing the rationale for this dual targeting strategy in vitro [33, 34].

Both M231 and 4T1 GPX4i‐resistant lines had increased resistance to combined inhibition of FSP1 (with viFSP1, 4T1 and M231 lines) or FSEN1 (M231 line only) and GPX4 (with RSL3, ML210) treatment in vitro (Figure S2). GPX4i‐resistant 4T1 cells, but not M231 cells, were more sensitive than the parental cells to the dual inhibition of xCT or GCLC (with BSO) and FSP1. Because M231 RSL3^R^ lines exhibited increased xCT expression, whereas 4T1 RSL3^R^ lines did not (Figure 1E,F), the lack of additional sensitivity to GCLC inhibition in M231 cells suggests that intracellular glutathione pools are not limiting in this line. This provides a potential explanation, which remains to be tested, for the observed differences between the 4T1 and M231 lines and may also explain why drug‐holiday cells remain resistant to Erastin2 despite xCT expression returning to parental levels, as glutathione pools may remain sustained. Together, these findings indicate that GPX4i resistance confers broad tolerance to dual ferroptosis pathway targeting, while revealing cell line‐specific differences that may underlie persistent resistance even after drug withdrawal.

### 2.3 GPX4‐Inhibitor Resistant and Parental Cells Maintain Similar Proliferation Rates In Vitro

We next evaluated the proliferation rate and cell cycle profile of the parental versus GPX4i‐resistant lines in vitro. No significant differences in proliferation rates were observed in the GPX4i‐resistant cells compared to the parental lines, as assessed by live‐imaging of cell confluency, or by cell cycle status, as measured by BrdU incorporation (Figure S3A–F). To test the necessity of GPX4 for GPX4i‐resistant cell survival, we used CRISPR/Cas9 to knockout *Gpx4* in the RSL3^R^ and parental 4T1 lines (Figure S3G). Cells were initially maintained in culture with 1 µm liproxstatin‐1 (Liprox), as *Gpx4* knockout is generally lethal for cells in vitro in the absence of a lipid peroxidation inhibitor. Upon Liprox removal, RSL3^R^ and parental 4T1 cells had similar proliferation rates and cell viabilities as assessed by live‐imaging and SYTOX‐green staining to measure cell death, indicating that GPX4 was still necessary for cell survival in the GPX4i‐resistant cell lines (Figure S3H,I).

### 2.4 Elevation of Lipid Peroxidation in GPX4‐inhibitor resistant Lines Is Reversible In Vitro

Because reactive oxygen species (ROS) and lipid peroxidation limit cancer cell survival in the blood, and antioxidant defenses are critical for metastasis [22, 23, 39, 40], we next assessed whether the GPX4i‐resistant cells had altered redox balance. We measured lipid peroxidation (BODIPY‐C11), mitochondrial/nuclear ROS (CellROX‐Green), and cytosolic ROS (CellROX‐DeepRed) by flow cytometry. Lipid peroxidation was elevated in RSL3^R^ and ML210^R^ cells compared to parental lines, but this increase was not observed in the drug‐holiday (DH) lines, further emphasizing the reversible nature of the resistance phenotype (Figure 1G,H; Figure S4D,E). Cytosolic and mitochondrial/nuclear ROS remained unchanged in the RSL3^R^ compared to parental cells, while they decreased in the ML210^R^ line after ML210 withdrawal (Figure S4F,G).

### 2.5 GPX4‐Inhibitor Resistant and Parental Cells Maintain Similar Redox States In Vitro

To further assess redox status, we measured the key indicators of redox homeostasis: reduced glutathione to oxidized glutathione ratios (GSH/GSSG) and NADP+/NADPH ratios. Consistent with our earlier findings that M231 RSL3^R^ cells failed to show additional sensitivity to GCLC inhibition, suggesting that glutathione availability may not be limiting in these lines, GSH and GSSG levels were higher in M231 RSL3^R^ only (Figure S4H–J). However, no differences in GSH/GSSG ratios between the GPX4i‐resistant and parental cells were observed, indicating maintenance of redox homeostasis (Figure 1I; Figure S4H–J). Similarly, NADP^+^ and NADPH levels trended toward a reduction in GPX4i‐resistant derived from 4T1, but not M231, cells (Figure 1I,J; Figure S4K–M). Collectively, these results show that GPX4i‐resistant cells maintain normal redox balance and proliferation in vitro, with reversible changes in lipid peroxidation after drug withdrawal. Given the limited changes observed in vitro, we next sought to characterize features present in in vivo contexts (including, but not limited to, oxidative stress, nutrient fluctuations, and immune interactions), that could potentially reveal biologically actionable adaptations conferring differences in metastatic potential in the GPX4i‐resistant lines.

### 2.6 GPX4‐Inhibitor Resistant Tumors Have Decreased Primary Tumor Growth

**FIGURE 2:**
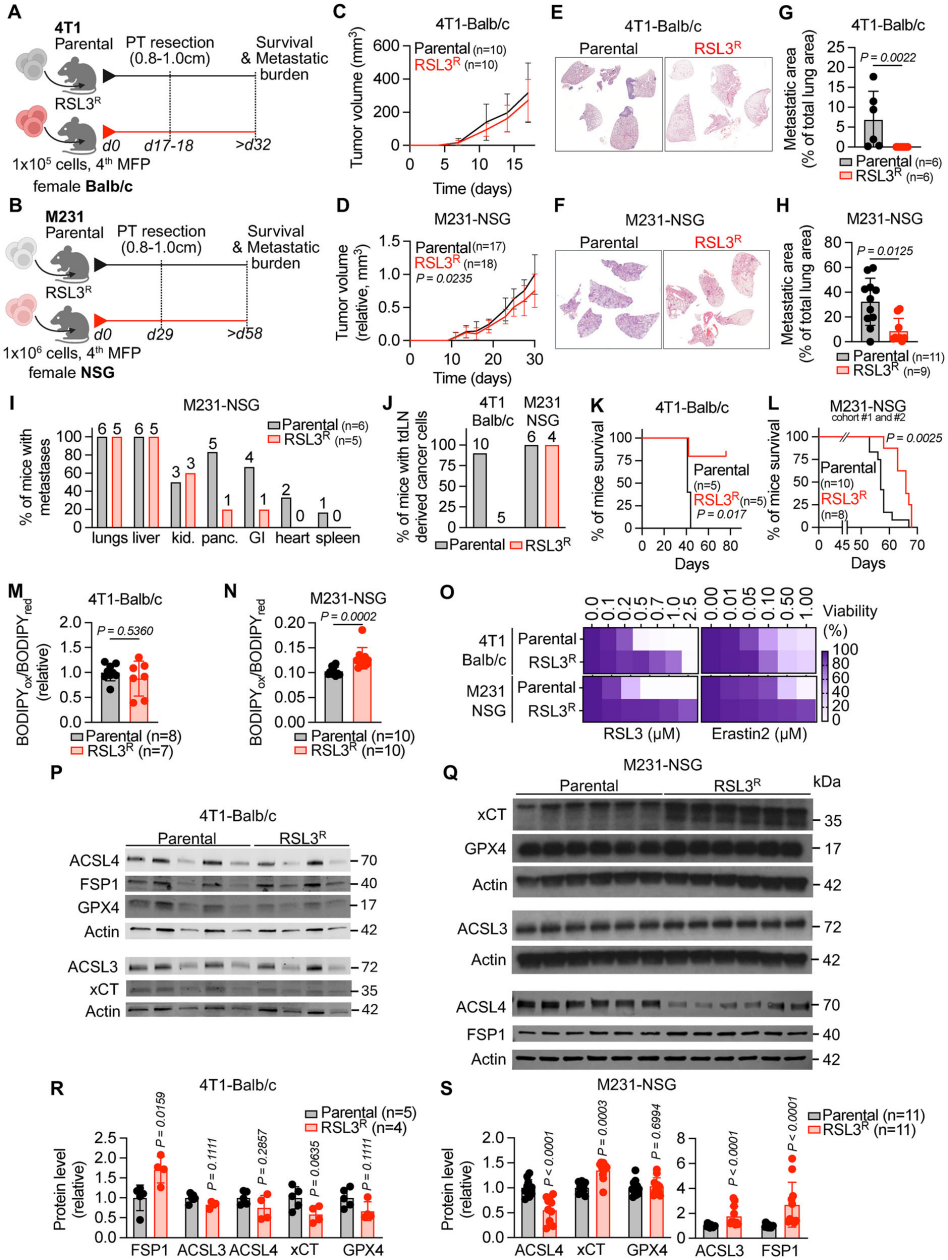
GPX4‐inhibitor resistant tumors form fewer distant metastases. (A,B) Experimental scheme of the 4T1‐Balb/c (A) and M231‐NSG (B) model of spontaneous metastasis from a surgically removed primary tumor (*n* = 2 independent experiments per model). (C,D) Tumor growth curves of 4T1‐Balb/c (C) and M231‐NSG (C) models (*n* = 7–18 mice per group). (E–H) Representative images of lung sections stained with hematoxylin and eosin (H&E) from 4T1‐Balb/c (E) and M231‐NSG (F) models. Quantification of the percentage of the tumor burden area covering the lung is shown in (G,H) (*n* = 6–11 mice per group). (I) Percentage of mice with distant metastases visually quantified in lungs, liver, kidneys (kid.), pancreas (panc.), GI tract, heart, and spleen in M231‐NSG model (*n* = 5–6 mice per group). (J) Percentage of mice with cancer cells isolated from tdLNs in 4T1‐Balb/c and M231‐NSG models (*n* = 4–10 mice per group). (K,L) Survival curves of 4T1‐Balb/c (K) and M231‐NSG (L) models (*n *= 5–10 mice per group). (M,N) Lipid peroxidation levels quantified by C11‐BODIPY 581/591 staining in cancer cells isolated from the resected primary tumor in the 4T1‐Balb/c model (M) and in the M231‐NSG model (N) (*n* = 7–10 mice per group). (O) Cancer cells were isolated from the resected primary tumor in 4T1‐Balb/c and M231‐NSG models and incubated with dose‐range RSL3 and Erastin2 for 48 h. Cell viability was assessed using an absorbance‐based assay (MTT), and results are expressed as a ratio to the untreated condition (*n* = 6–8 mice per group). (P–S) Immunoblot analysis of protein extracts from cancer cells isolated from the resected primary tumor in 4T1‐Balb/c (P) and M231‐NSG (Q) using the indicated antibodies. Protein level quantification relative to the average of parental control group are represented in (R,S) (*n* = 4–11 mice per group). Data in (C,D) are displayed as mean ± s.d. and were analyzed by two‐way ANOVA and Tukey's multiple comparation. Data in (G,H,M,N,R,S) are displayed as mean ± s.d. and were analyzed by two‐sided Mann–Whitney test. Kaplan–Meyer plots in (K,L) were analyzed by the Mantel–Cox log‐rank test. Data in (O) are displayed as the mean.

To investigate the in vivo impact of GPX4i‐resistant cells on tumor growth and metastasis, we orthotopically (into the mammary fat pad) transplanted parental and RSL3^R^ cells derived from the 4T1 and M231 cell lines into Balb/c and NSG mice, respectively. Once the tumors reached 1.0 cm in size, we performed survival surgeries in which we removed the primary tumor completely to allow for metastatic outgrowth of the disseminated cancer cells that would otherwise be limited by the primary tumor outgrowth (Figure 2A,B). Throughout the duration of the experiment, no significant changes in the body weight of mice were observed (Figure S5A–D). The tumor growth rate of the RSL3^R^ tumors was similar to that of the parental tumors prior to surgical removal of the tumor in the 4T1‐Balb/c model (Figure 2C; Figure S5E). In the M231‐NSG model, the tumor growth rate of the RSL3^R^ tumors was slightly, but significantly, decreased compared to the parental tumors (Figure 2D; Figure S5F).

In a second experiment focused on primary tumor growth, tumors were grown to 1.5 cm without resection, unlike the survival surgery model, where tumors are removed at ∼1 cm. Under these conditions, RSL3^R^ and ML210^R^ tumors grew more slowly than parental tumors in the 4T1‐Balb/c model (Figure S5G). While growth rates were similar across groups for the first 20 days, ML210^R^ tumors later slowed, and half of the RSL3^R^ tumors regressed (Figure S5H,I). BrdU labeling and flow cytometry showed no differences in cell cycle profiles between RSL3^R^ and parental tumors (Figure S5J), indicating that reduced proliferation does not explain the observed tumor growth differences. These findings point to non‐proliferative factors, such as impaired cell survival or enhanced immune clearance, among other possibilities, as drivers of the reduced tumor growth observed in the GPX4i‐resistant tumors.

### 2.7 GPX4‐Inhibitor Resistant Tumors Form Fewer Distant Metastases

Since the lungs are the primary site of spontaneous metastasis from the mammary fat pad in both 4T1‐Balb/c and M231‐NSG models, we quantified lung metastases after surgical resection of the primary tumors (Figure 2A,B). Mice with RSL3^R^ tumors developed significantly fewer lung metastases than mice with parental tumors in both models (Figure 2E–H), independent of differences in primary tumor size (Figure S5K–L).

In the M231‐NSG model, which permits broader dissemination of metastases due to the immune deficiency of the NSG mice, we observed fewer visible metastases in distant organs (i.e., liver, kidney, pancreas, and GI tract) in RSL3^R^ tumor‐bearing mice (Figure 2I). Tumor cells were detected in the tumor‐draining lymph nodes (tdLNs) in both parental and RSL3^R^ mice in the M231‐NSG model, but in the 4T1‐Balb/c model, tdLN involvement was only seen in parental tumor‐bearing mice (Figure 2J). This reduced metastatic burden translated into a marked survival benefit: Balb/c mice with RSL3^R^ tumors remained healthy and metastasis‐free through day 80, the endpoint of the survival study (Figure 2K). This survival advantage was recapitulated in the M231‐NSG model in which mice with RSL3^R^ tumors showed a median survival of 66 days versus 57 days for mice with parental tumors (Figure 2L). Notably, lipid peroxidation levels in primary tumor cells were similar across groups in the 4T1‐Balb/c model (Figure 2M) and were slightly increased in cancer cells derived from RSL3^R^ tumors compared to parental tumors in the M231‐NSG model (Figure 2N).

Given that the lipid peroxidation levels in the primary tumor cells did not differ substantially across groups in vivo, we next sought to test the extent to which our GPX4i‐resistant lines maintained their resistance to ferroptosis in vivo. To test this, we isolated RSL3^R^ cells from primary tumors of the tumor‐bearing mice and treated them ex vivo with RSL3 and Erastin2. Cancer cells isolated from RSL3^R^ tumors compared to parental tumors in both 4T1‐Balb/c and M231‐NSG models had increased survival upon ex vivo RSL3 and Erastin2 treatment, thus confirming maintenance of resistance to ferroptosis‐induction in the RSL3^R^ lines in vivo (Figure 2O; Figure S5M–P). In these ex vivo cancer cells, however, we observed varying changes in protein levels of key ferroptosis regulators; FSP1 was notably increased in both the 4T1‐Balb/c and M231‐NSG mice in cells isolated from the RSL3^R^ compared to parental tumor‐bearing mice, which could possibly account for the maintained lipid peroxidation levels, though this remains to be directly tested (Figure 2P–S; Figure S5Q).

### 2.8 GPX4i‐Resistant and Parental TNBC Cells Display Similar Immune Infiltration Profiles

**FIGURE 3:**
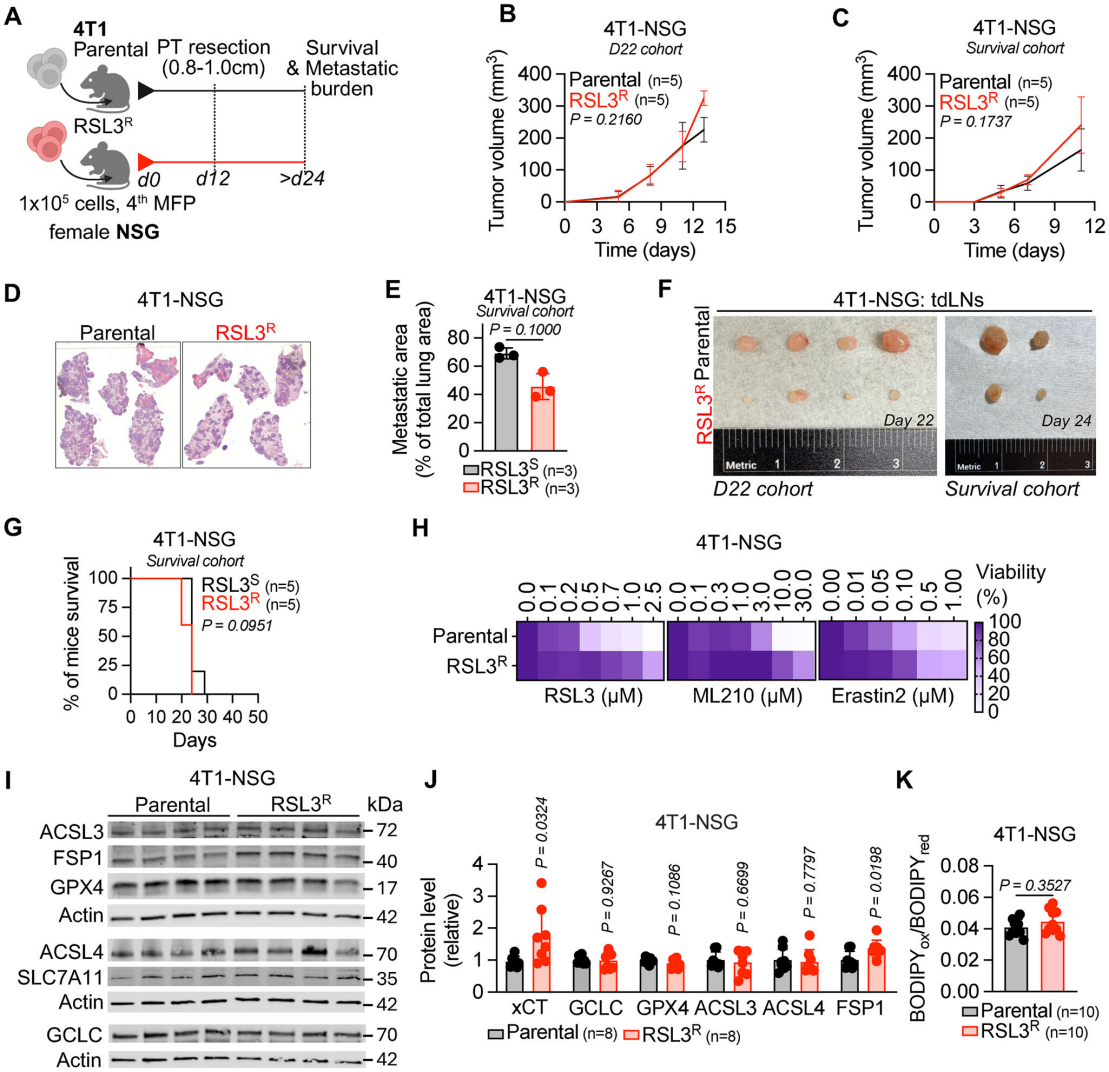
GPX4i‐resistant 4T1 tumors form fewer distant metastases in immunocompromised NSG mice. (A) Experimental scheme of the survival surgery model of the 4T1‐NSG model (*n* = 2 independent experiments). (B,C) Tumor growth curve of D22 cohort (B) and Survival cohort (C) in 4T1‐NSG model (*n* = 5 mice per group). (D,E) Representative images of lung sections stained with hematoxylin and eosin (H&E) from 4T1‐NSG (D) models. Quantification of the percentage of the tumor burden area covering the lung is shown in (E) (*n* = 3 mice per group). (F) tdLNs size from 4T1‐NSG model (*n* = 6 mice per group). (G) Survival curves of 4T1‐NSG model (*n* = 5 mice per group). (H) Cancer cells were isolated from the resected primary tumor from D22 and Survival cohorts in the 4T1‐NSG model and incubated with dose‐range RSL3, ML210, and Erastin2 for 48 h. Cell viability was measured using an absorbance‐based assay (MTT), and results are expressed as a ratio to the untreated condition (*n* = 10 mice per group). (I,J) Immunoblot analysis of protein extracts from cancer cells isolated from the resected primary tumor in 4T1‐NSG using the indicated antibodies (I). Protein level quantification relative to the average of the parental control group is represented in (J) (*n* = 8 mice per group). (K) Lipid peroxidation quantified by C11‐BODIPY 581/591 staining in cancer cells from the resected primary tumors of the D22 cohort in the 4T1‐NSG model (*n* = 10 mice per group). Data in (B,C) are displayed as mean ± s.d. and were analyzed by two‐way ANOVA and Tukey's multiple comparison. Data in (E,J,K) are displayed as mean ± s.d. and were analyzed by two‐sided Mann–Whitney test. Kaplan–Meyer plot in (G) was analyzed by Mantel–Cox log‐rank test.

Because half of the RSL3^R^ tumors regressed in the immunogenic 4T1‐Balb/c model (Figure S5H), we next investigated whether 4T1 RSL3^R^ tumors would also show reduced metastatic burden when injected into immunocompromised NSG mice. We orthotopically transplanted 4T1 parental and RSL3^R^ cells in NSG mice using the same batch of cells used in the 4T1‐Balb/c model and surgically resected the tumors at 1 cm to allow time for metastasis development (Figure 3A). One cohort was used to quantify the metastatic burden at a time‐matched point (Day 22, ‘D22’ cohort), and another cohort was used for survival analysis (‘Survival’ cohort). Tumor growth and body weight were similar in mice bearing RSL3^R^ or parental tumors (Figure 3B,C; Figure S6A–E). Despite this, mice bearing RSL3^R^ tumors tended to have fewer metastases, which was independent of primary tumor size (Figure 3D–F; Figure S6G). However, there were no significant survival differences between mice bearing tumors with the RSL3^R^ or parental lines in the 4T1‐NSG model (Figure 3G). Although immunocompetent Balb/c mice bearing 4T1 RSL3^R^ cells had decreased spontaneous metastases to the lung, a decreased presence of cancer cells in tumor‐draining LNs, and an increased overall survival, these effects were not observed in immunocompromised NSG mice bearing 4T1 RSL3^R^ tumors.

We next tested whether 4T1 RSL3^R^ cells retain ferroptosis resistance in NSG mice, as seen in Balb/c mice. Cancer cells isolated from resected tumors remained resistant to RSL3, ML210, and Erastin2 ex vivo (Figure 3H; Figure S6H–J). FSP1 protein levels were also elevated in RSL3^R^ tumors from 4T1‐NSG mice (Figure 3I,J). Notably, xCT expression was increased in RSL3^R^ tumors from NSG mice but decreased in RSL3^R^ tumors from Balb/c mice. No changes were observed in ACSL3, ACSL4, GCLC, or lipid peroxidation levels in the 4T1‐NSG model (Figure 3I–K).

Since delayed tumor growth and improved survival were observed in Balb/c mice, but not NSG mice, bearing tumors from 4T1 RSL3^R^ or ML210^R^ cells, we next profiled the immune populations within these different tumor models. We transplanted 4T1 parental and RSL3^R^ cells into Balb/c mice, collected tumors at 1 cm, and profiled immune cells by flow cytometry. No significant differences in the absolute cell counts of immune cells (CD45+), T cells (CD3+, including CD4+ cells and CD8a+ cells), dendritic cells (DCs, CD11c+), macrophages (F4/80+), pro‐tumorigenic macrophages (CD163+), neutrophils (Gr1+), and B cells (B220+) were observed (Figure S7A–I). MHCII expression on CD45+ cells and activation markers (IFNγ, PD‐1) on CD8+ T cells were also slightly increased or unchanged, respectively (Figure S7J–L).

### 2.9 GPX4i‐Resistant Tumors Show Distinct Lipid Adaptations in the Immunocompetent Model

**FIGURE 4:**
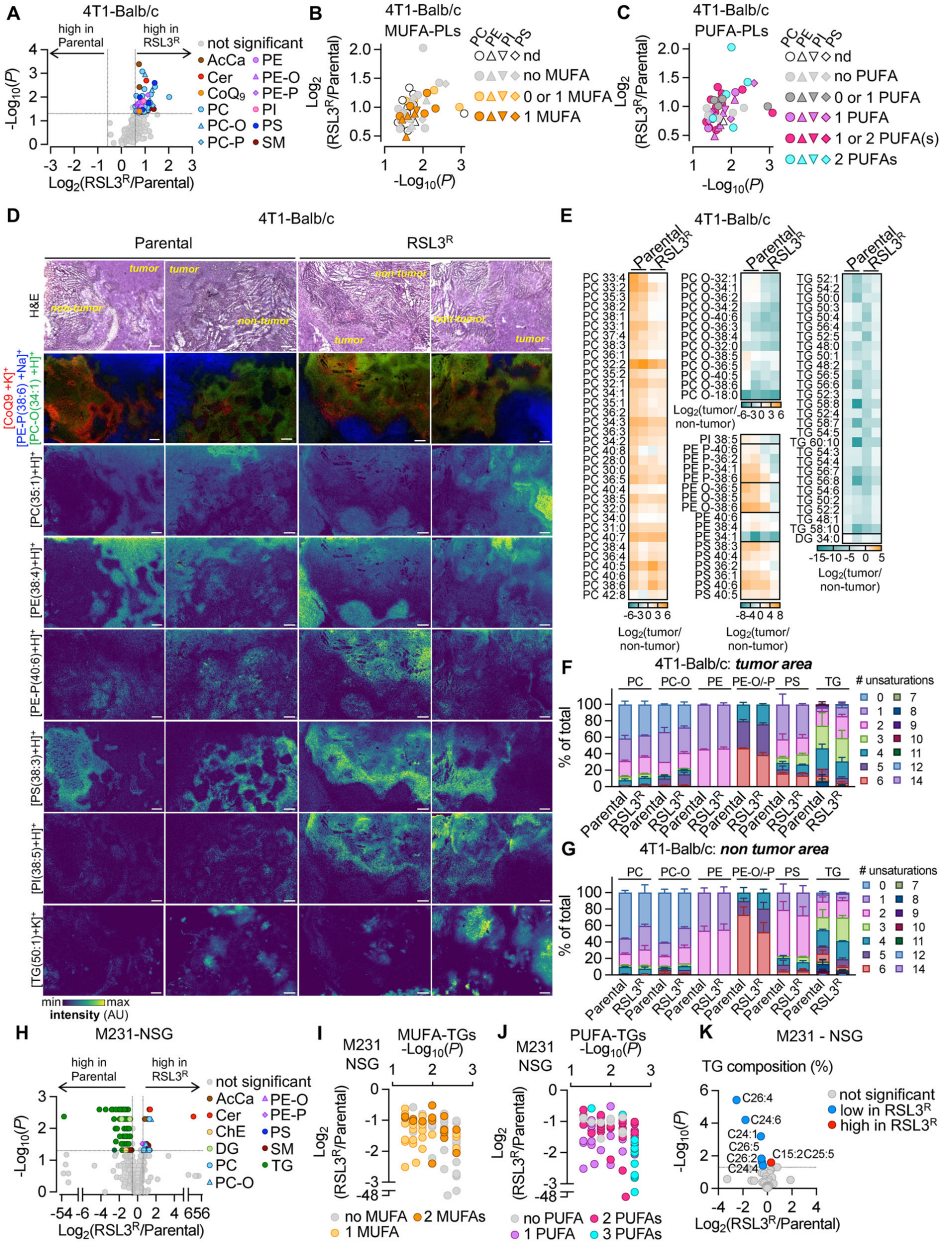
GPX4i‐resistant tumors show distinct lipid adaptations in the immunocompetent model. (A–C) Lipid profiling was performed on cancer cells isolated from the resected primary tumor in 4T1‐Balb/c by LC/MS (*n* = 7 mice per group). FC of lipid peak intensities and *p*‐value for RSL3^R^‐ versus parental‐derived cancer cells comparisons calculated using MetaboAnalyst are shown in (A), and significant changes in lipid peak intensities are highlighted (FC ≥ 1.5 and ≤0.66, *p* < 0.05). MUFA and PUFA composition of significantly increased phospholipids are represented in (B,C) respectively. (D–G) Spatial lipid profiling was performed on the resected primary tumor in 4T1‐Balb/c by MALDI‐MSI (*n* = 2 mice per group). Images of primary tumor sections stained with hematoxylin and eosin (H&E) and the corresponding spatial distribution of representative lipid species from the same section are shown in (D) (scale bar = 250 µm). FC of lipid peak intensities in tumor versus non‐tumor areas is represented in (E). Unsaturation levels are represented in percent for each lipid class in tumor areas (F) and in non‐tumor areas (G). (H–K) Lipid profiling was performed on cancer cells isolated from the resected primary tumor in M231‐NSG by LC/MS (*n* = 5–7 mice per group). FC of lipid peak intensities and *p*‐value for RSL3^R^‐ versus parental‐derived cancer cells comparisons calculated using MetaboAnalyst are shown in (H), and significant changes in lipid peak intensities are highlighted (FC ≥ 1.5 and ≤0.66, *P* < 0.05). MUFA and PUFA composition of significantly decreased triglycerides are represented in (I,J) respectively. Fatty acyl chain composition of triglycerides is represented in (K). AcCa: acyl carnitine, Cer: ceramides, ChE: cholesterol ester, CoQ_9_: coenzyme Q9, PC(‐P/‐O): phosphatidylcholine (‐plasmalogen/‐alkyl ether), PE(‐P/‐O): phosphatidylethanolamine (‐plasmalogen/‐alkyl ether); PI: phosphatidylinositol; PS: phosphatidylserine, PUFA: polyunsaturated fatty acids, MUFA: monounsaturated fatty acids, SM: sphingomyelin, TG: triglycerides.

We next characterized the metabolomic and lipidomic profile of the GPX4i‐resistant tumors resulting from the NSG versus Balb/c in vivo. We profiled the metabolic and lipid composition of cancer cells from resected primary tumors in 4T1‐Balb/c, 4T1‐NSG, and M231‐NSG models (Figure 4A–K; Figures S8A–C, S9A–L, S10A–J, S11A–G, and S12A–N).

In the Balb/c mice, cancer cells isolated from RSL3^R^ tumors displayed an increase in total fatty acids (Figure S8A). This increase in fatty acid levels was associated with an increase in total phospholipid (PE‐P, PC, PC‐P, PI, and PS) content, along with elevated Coenzyme Q9 (CoQ_9_), a known substrate of FSP1 as measured by LC/MS (Figure 4A; Figure S9A–C). Cell size remained unchanged, ruling out increased membrane area as a cause of higher phospholipid content (Figure S9D). These lipidomic shifts suggest an adaptive lipid remodeling in the Balb/c mice which could indirectly contribute to increased ferroptosis resistance. We next investigated if GPX4i‐resistant tumors had reduced PUFA content, given that PUFAs are prone to lipid peroxidation [38, 41]. No major changes were observed in the MUFA or PUFA content of the phospholipids (Figure 4B,C) or triglycerides of the significantly changed lipid classes in tumors from the GPX4i‐resistant versus parental lines in the 4T1‐Balb/c model (Figure S9E–L). In contrast, RSL3^R^ compared to parental tumors from the NSG mice showed no significant differences in lipid profiles, but had significant changes in metabolites associated with glutathione, arginine, and one‐carbon metabolism (glycine, serine, and threonine metabolism and cysteine and methionine metabolism) (Figures S8B and S10A–J).

To distinguish whether the lipid changes in the 4T1 models arose from tumor cells or from infiltrating immune cells in the 4T1 model, we next sought to conduct spatial profiling of lipid composition of resected primary tumor in 4T1‐Balb/c mice using MALDI‐MSI (Figure 4D). H&E staining of the corresponding primary tumor sections was used to differentiate the lipid composition of the microenvironment (non‐tumor areas) from the tumor areas (Figure 4D; Figure S11A). Lipid annotations from MALDI‐MSI were cross‐validated with the lipid annotations obtained on the same samples by LC/MS (Figure 4A). This approach revealed that tumor areas and non‐tumor areas can be differentiated based on their spatial lipid profiling (Figure 4D). For example, the PE‐P(38:6) signal was higher in tumor areas, while the PC‐O(34:1) was higher in non‐tumor areas. Interestingly, the CoQ_9_ signal increased at the periphery between tumor and non‐tumor areas (Figure 4D).

Comparison of the enriched lipid species in tumor versus non‐tumor areas showed distinct lipid profiles in RSL3^R^ and parental tumor regions. While PCs were higher in tumor areas, especially from parental primary tumors, PC‐Os showed reduced signal in tumor areas from RSL3^R^ primary tumors (Figure 4E). Distinct lipid distributions were observed for other phospholipids including PI, PE‐P, and PE‐O (Figure 4E). However, TGs were consistently lower in tumor areas from both parental and RSL3^R^ primary tumors (Figure 4E). We next investigated the extent to which the spatial distribution of phospholipids and triglycerides based on the saturation content of the fatty acids was associated with the presence of RSL3^R^ tumors. Phospholipids and triglycerides detected in the non‐tumor areas versus tumor areas from the RSL3^R^ compared to the parental tumors tended to contain fatty acid chains bearing fewer degrees of unsaturation (Figure 4F,G; Figure S11B–G). Together, these results demonstrate tumors derived from the RSL3^R^ versus parental lines in immunocompetent mice have unique lipidomic profiles.

Last, RSL3^R^ tumors in NSG mice transplanted with human M231 cells showed both distinct metabolic and lipid shifts associated with an increase in metabolites related to UDP‐sugar metabolism and a significant decrease in metabolites related to purine metabolism (Figure S8C). In contrast to 4T1‐Balb/c mice (Figure S8A), cancer cells isolated from RSL3^R^ tumors from M231‐NSG mice displayed a decrease in total fatty acid content (Figure S8C). This decrease in fatty acid levels was associated with a decrease in total triglyceride content while fewer increases were observed in phospholipids, and no changes were observed in CoQ levels (Figure 4H; Figure S12A–E). There were no significant differences in cancer cell size in RSL3^R^ versus parental tumors in the M231‐NSG model (Figure S12F). Among the significantly decreased TGs, those containing fatty acid chains with 3 PUFAs were the most reduced in the RSL3^R^ tumor compared to the parental tumor (Figure 4I,J). This was associated with a reduction in total long‐chain fatty acid content within total triglycerides in RSL3^R^ tumor compared to parental tumor, but not in total phospholipids (Figure 4K; Figure S12H–N).

Collectively, these findings emphasize variations in metabolic and lipid adaptations across tumor models and microenvironments. The extensive differences in the metabolomic and lipidomic profiles of the GPX4i‐resistant compared to primary tumors in immunocompromised versus immunocompetent models further emphasize the context‐dependency of studying ferroptosis resistance in vivo.

### 2.10 GPX4i‐Resistant TNBC Cells Can Survive in Blood and Colonize Lungs

**FIGURE 5:**
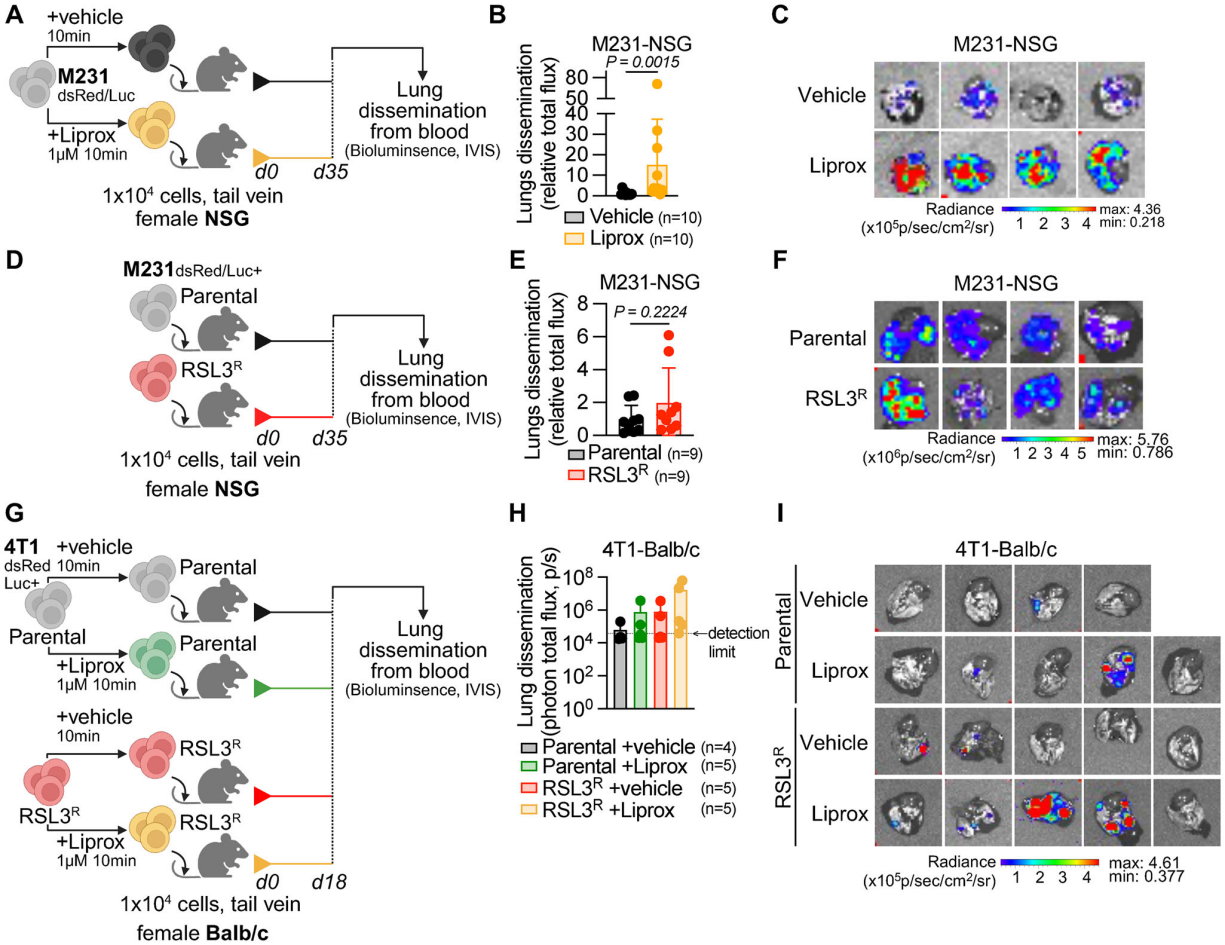
GPX4i‐resistant TNBC cells can survive in the blood and colonize the lungs. (A) Experimental schematic of breast cancer cell survival and colonization in lungs of NSG mice after intravenous injection with M231 breast cancer cells pre‐treated with vehicle or with Liprox (*n* = 2 independent experiments). (B,C) Quantification of breast cancer cell survival and colonization in lungs (B) based on *ex vivo* bioluminescence imaging (C) in NSG mice intravenously injected with M231 cells treated with vehicle or Liprox. (D) Experimental schematic of breast cancer cell survival and colonization in lungs of NSG mice after intravenous injection with M231 parental or M231 RSL3^R^ breast cancer cells (*n* = 2 independent experiments). (E,F) Quantification of breast cancer cell survival and colonization (E) based on ex vivo bioluminescence imaging (F) in NSG mice intravenously injected with parental or RSL3^R^ cells derived from the M231 cell line. (G) Experimental schematic of breast cancer cell survival and colonization in lungs of Balb/c mice after intravenous injection with 4T1 parental or 4T1 RSL3^R^ cells pre‐treated with vehicle or with Liprox (*n* = 1 experiment). (H,I) Quantification of breast cancer cell survival and colonization (H) based on ex vivo bioluminescence imaging (I) in Balb/c mice intravenously injected with parental or RSL3^R^ cells derived from the 4T1 cell line and treated with vehicle or liprox. Data in (B,E,H) are displayed as mean ± s.d. and were analyzed by two‐sided Mann–Whitney test.

Prior work has shown that ROS and lipid peroxidation limit cancer cell survival in the blood in melanoma murine models [23, 24], but this has not yet been assessed in breast cancer models. To test this, we pre‐treated dsRed/luciferase‐tagged M231 cells for 10 min with or without Liprox, an antioxidant that traps lipid peroxyl radicals to inhibit ferroptotic cell death, or vehicle. Then, we transplanted the cells by intravenous injection in the tail vein of NSG mice and measured cancer cells dissemination to lungs by bioluminescence detection of luciferase activity (Figure 5A). Similar to the prior findings in melanoma models [4], in the M231 breast cancer model Liprox pre‐treatment increased the formation of lung metastasis after intravenous injection (Figure 5B,C). This result suggests that lipid oxidation is also limiting breast cancer cell survival in the blood.

Next, we hypothesized that although GPX4i‐resistant cells form fewer spontaneous metastases from orthotopic tumors, they may still have a survival advantage against lipid oxidative stress in the bloodstream. To test this, we injected dsRed/luciferase‐tagged RSL3^R^ and parental M231 cells intravenously into NSG mice and monitored lung colonization by bioluminescence imaging (IVIS) of the luciferase‐tagged cancer cells (Figure 5D). M231 RSL3^R^ cells had similar lung colonization compared to parental cells, indicating both lines had similar survival in the blood after intravenous injection (Figure 5E,F).

We then tested whether 4T1‐derived RSL3^R^ cells, which failed to form detectable metastases in the spontaneous Balb/c model, could still survive in circulation when injected directly into the bloodstream. After intravenous injection, RSL3^R^ cells colonized the lungs at levels similar to parental cells, indicating comparable survival in the blood (Figure 5G–I). These findings suggest that the lack of spontaneous metastasis in RSL3^R^ tumors is not due to impaired survival in circulation, but may reflect a reduced ability to extravasate or establish secondary tumors. To test if lipid oxidation limits 4T1‐derived RSL3^R^ cells' survival in blood, we transplanted Liprox‐pretreated RSL3^R^ and parental cells intravenously into Balb/c mice. Liprox pre‐treatment and subsequent intravenous injection of parental or RSL3^R^ cells had a tendency toward increased lung metastases in both Liprox‐treated cohorts (Figure 5G–I). These results suggest that, despite their acquired resistance to GPX4i, RSL3^R^ cells remain vulnerable to lipid oxidation during hematogenous dissemination.

Since we had performed analyses at the protein, metabolomic, and lipidomic levels, we next performed RNA sequencing on parental, RSL3^R^, RSL3^R^ DH, ML210^R^, and ML210^R^ DH lines derived from 4T1 and M231 cells in vitro to investigate if there may be transcriptional changes that could explain why GPX4i‐resistant tumors showed a reduced capacity to form spontaneous metastases (Table S1). Using gene set enrichment analysis (GSEA), we identified common gene signatures that were either enriched or depleted in GPX4i‐resistant lines maintained with RSL3 or ML210 and upon drug withdrawal (Figure S13A–C; Table S1). Among the depleted signatures, those associated with epithelial‐mesenchymal transition (EMT) were prominent. Interestingly, GPX4 dependency has been described as a feature of mesenchymal breast cancer cells linking EMT plasticity to ferroptosis vulnerability [12, 29, 42]. In line with the transcriptomic profiling, the protein levels of the mesenchymal marker vimentin were lower consistently reduced in GPX4i‐resistant lines (Figure S13D–G). This reduction was confirmed in cancer cells isolated from the RSL3^R^ tumor from the M231‐NSG model, whereas no changes were observed in cancer cells isolated from the RSL3^R^ tumor from the 4T1‐NSG model (Figure S13H–J).

Conversely, protein levels of the epithelial marker E‐cadherin were significantly higher in RSL3^R^ derived from 4T1 cells, specifically in tumors from the NSG mice (Figure S13D; Figure S13G,H,J). Similarly, the epithelial marker EpCAM was consistently elevated at the cell surface of RSL3^R^ compared to parental tumor cells in 4T1‐Balb/c, M231‐NSG, and 4T1‐NSG models (Figure S13K). Together, these results indicate that GPX4i‐resistant tumors exhibit a more epithelial‐like phenotype which may contribute to their decreased capacity to form spontaneous metastasis as more epithelial‐like cells are less able to intravasate to form distant metastases [43].

## 3 Discussion

**FIGURE 6:**
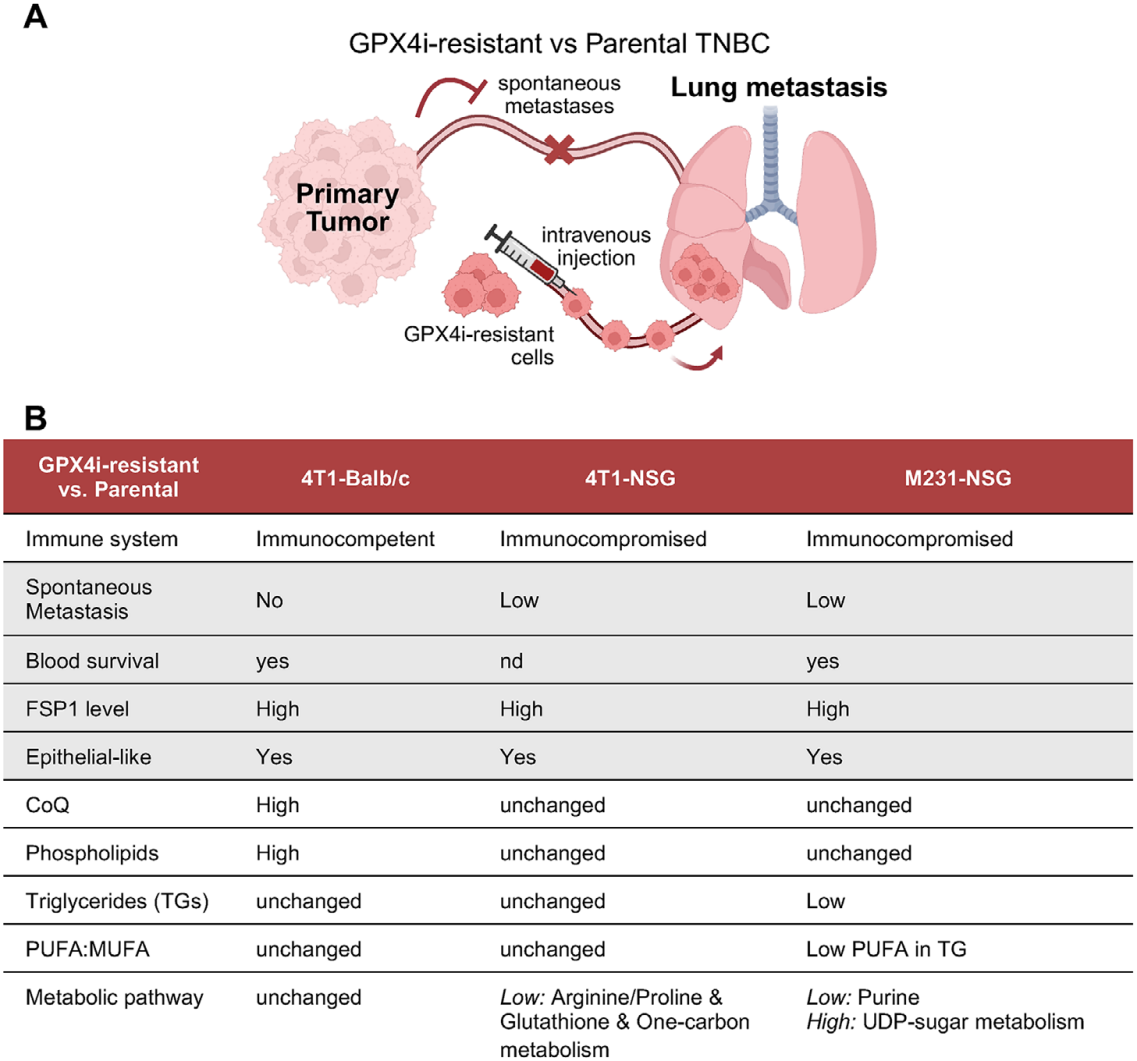
Summary of the adaptations associated with GPX4 inhibitor resistance in vivo. (A) Schematic diagram recapitulating the common identified adaptation associated with GPX4 inhibitor resistance between in vivo models. GPX4i‐resistant tumor showed an epithelial‐like state and increased levels of FSP1. Spontaneous metastases are reduced in GPX4i‐resistant tumor whereas GPX4i‐resistant cancer cells survive in the blood and can colonize lungs when transplanted in the bloodstream (created in BioRender. Sabatier, M. (2026) https://BioRender.com/sy8r3mn). (B) Table summarizing the context‐dependent adaptations identified GPX4i‐resistant tumors compared to parental tumors in each murine model.

In this study, we characterized how resistance to GPX4 inhibition in vitro impacts metastatic features in TNBC in vivo. Here, we observed that GPX4i‐resistant tumors have reduced metastatic potential in immunocompetent mice, although they retain the ability to survive in the bloodstream and colonize distant sites upon direct intravenous injection (Figure 6A). These findings suggest that resistance to small‐molecule GPX4 inhibitors may be associated with altered metastatic potential, particularly during the early steps of the metastatic cascade. Tumors from GPX4i‐resistant compared to parental cell lines behaved differently in immunocompetent versus immunodeficient mice, with clear differences in lipid and metabolic rewiring (Figure 6B). A key implication of these findings, which remains to be mechanistically explored in future studies, is that the microenvironment influences the in vivo profile of tumors formed from GPX4i‐resistant cell lines.

The observation that GPX4i‐resistant cells can survive in circulation but fail to form spontaneous metastases raises the possibility that resistance to GPX4 inhibitors may interfere with EMT or other traits necessary for early metastatic escape [19, 44]. Prior work has shown that mesenchymal phenotypes are more sensitive to ferroptosis, particularly in breast cancer models, and that transcription factors like ZEB1 modulate ferroptosis sensitivity through EMT plasticity [12, 29, 42]. Accordingly, we show that resistance to GPX4 inhibitors is associated with a more epithelial‐like state in our breast cancer models, which may protect the cancer cells from lipid peroxidation but could simultaneously reduce their capacity to intravasate and form spontaneous metastases. This provides a plausible mechanistic possibility, which remains to be tested, for why GPX4i‐resistant cells retain viability in the blood, yet are unable to efficiently form spontaneous metastasis from a primary tumor.

Importantly, the reduced spontaneous metastatic capacity observed in the GPX4i‐resistant tumors should not be assumed to be a universal consequence of GPX4 inhibitor resistance, as the underlying mechanisms of resistance may have distinct biological consequences. For example, resistance driven by changes to ACSL4 may result in modifications to PUFA‐containing phospholipids and altered susceptibility to lipid peroxidation, which could plausibly impact metastatic fitness. In contrast, resistance mediated by upregulation of FSP1 may preserve ferroptosis resistance without necessarily altering membrane lipid composition, and may therefore have different implications for metastatic potential. Thus, in these models, reduced metastatic capacity likely reflects specific resistance‐associated adaptations rather than a universal consequence of GPX4 inhibitor resistance per se, though this remains to be directly tested in future studies.

Our study also provides a framework to reinterpret foundational in vitro work with GPX4 inhibitors. For example, many studies using RSL3 or ML210 have demonstrated differential sensitivity to ferroptosis across cancer cell types, often correlating with mesenchymal features, high PUFA content, or low antioxidant capacity [45]. Our in vivo data suggest that these correlations may be context‐dependent and influenced by environmental cues not captured in vitro. Similarly, the role of FSP1 in buffering lipid peroxidation seen in 2D culture is consistent with our in vivo findings that FSP1 and CoQ_9_ are upregulated in resistant tumors under immune and oxidative pressure. By anchoring our study in widely used ferroptosis tool compounds, our findings bridge cellular models and the physiological complexities of cancer metastasis in vivo and highlight the need for targeted studies using bioavailable inhibitors that consider the full range of tumor–host interactions.

One key limitation of our work is that many of our conclusions are intentionally based on RSL3^R^ models because much of the foundational work in the ferroptosis field has relied on RSL3, and although we included ML210^R^ cells to help control for off‐target effects of RSL3, additional studies using other GPX4 inhibitors and genetic models will strengthen the generalizability of our findings. While we assessed primary tumor growth of ML210^R^ cells and observed markedly slower growth than parental tumors, this difference makes direct comparison of metastatic burden challenging, and future studies using optimized models with matched initial tumor growth will be needed to address this. Furthermore, our resistance models were generated in vitro using small molecule inhibitors, and not genetic perturbations, and thus our conclusions are limited to interpretations pertaining to the pharmacological effects of GPX4 resistance and may not fully reflect how resistance develops in a native tumor environment. Finally, while we characterized the lipidomic, metabolomic, and immune profiles of GPX4i‐resistant tumors compared to parental tumors, further studies are needed to dissect potential causal mechanisms linking these adaptations to altered metastasis.

Collectively, these data demonstrate that long‐term maintenance with GPX4‐inhibitors in vitro leads to altered metastatic features in vivo. These findings refine our understanding of ferroptosis biology in vivo and caution that preclinical studies of ferroptosis‐inducing agents should carefully consider specific impacts on the different steps of the metastatic cascade.

## 4 Materials and Methods

### 4.1 Cell Lines

The human MDA‐MB‐231 (M231, Cat#HTB‐26, RRID: CVCL_0062) and murine 4T1 (Cat#CRL‐2539, RRID: CVCL_0125) cell lines were purchased from the American Type Culture Collection in July 2022 and November 2022, respectively. The ATCC cell bank provides authenticated cell lines by short tandem repeat (STR) profiling. The HEK 293T (RRID: CVCL_0063) cell line was gifted from the T. Muranen Laboratory (Beth Israel Deaconess Medical Center, Harvard Medical School). These cell lines were tested every 4 months for *Mycoplasma* contamination using the MycoAlert detection kit (Lonza, Cat#LT07‐218) and were negative for *Mycoplasma*. The murine 4T1 cell line was grown at 37°C with 5% CO_2_ in Roswell Park Memorial Institute Medium (RPMI) 1640 with 2 mm l‐glutamine supplemented with 10% heat‐inactivated fetal bovine serum (HI‐FBS) and 100 IU/mL penicillin and 100 µg/mL streptomycin. Human M231 and HEK 293T cell lines were grown at 37°C with 5% CO_2_ in Dulbecco's Modified Eagle Medium (DMEM) with 25 mm l‐glucose and 1 ml l‐pyruvate supplemented with 10% HI‐FBS and 100 IU/mL penicillin and 100 µg/mL streptomycin. Cultured cells were passaged every 2–3 days and maintained in an exponential growth phase at low passage number (10–12 passages maximum).

### 4.2 Reagents

1S,3R‐RSL3 (RSL3) (Cat#HY‐100218A), ML210 (Cat#HY‐100003), Erastin2 (Cat#HY‐139087), FSEN1 (Cat#HY‐153629) and viFSP1 (Cat#HY‐163002) were purchased from MedChem Express. Liproxstatin‐1 (Liprox, Cat#17730) was purchased from Cayman Chemical. l‐Buthionine‐(S,R)‐sulfoximine (BSO, Cat#B2515) was purchased from Sigma‐Aldrich. RSL3, Erastin2, viFSP1, and Liprox stock solutions were prepared in dimethyl sulfoxide (DMSO) at 10 mm. ML210 was dissolved in DMSO by sonication at 40 mm.

### 4.3 Generation of GPX4‐Inhibition Resistant TNBC Cell Lines

dsRed/luciferase‐tagged 4T1 and M231 cell lines were exposed to increasing dosages (from 50 nm to 1 µm) of RSL3 or (from 500 nm to 30 µm) ML210 over a three‐month period (from 30 to 35 passages) (Figure 1A). Briefly, RSL3 and ML210 were directly added into fresh media every Monday, Wednesday, and Friday when splitting the cells. The media supplemented with either RSL3 or ML210 was replaced on the same days, even if the cell line was experiencing cell proliferation arrest and/or cell death (from 90% to 10% of cell death) to maintain the selection pressure. The dosage level was increased every time no additional cell death was detected, and proliferation was restored upon treatment with either RSL3 or ML210 by brightfield microscopy to monitor for cell confluency and floating cells. When the maximal dose of RSL3 (1 µm) and ML210 (30 µm) was reached without inducing proliferation arrest and cell death, the cell lines were maintained and expanded for two additional weeks with the small molecule inhibitors. Then, the generated RSL3^R^ and ML210^R^ cell lines were frozen in HI‐FBS supplemented with 10% DMSO. After thawing, the RSL3^R^ and ML210^R^ cell lines were maintained for one week with 1 µm RSL3 and 30 µm ML210, respectively, to recover the complete resistance to these drugs (defined as proliferating cells, without cell death) before performing any in vitro or in vivo experiment. The obtained dsRed/luciferase‐tagged 4T1 Parental, 4T1 RSL3^R^, 4T1 ML210^R^, M231 Parental, and M231 RSL3^R^ were authenticated by STR profiling in April 2025. RSL3^R^ and ML210^R^ cell lines were subjected to a drug holiday during which cells were not exposed to the drug for two passages (i.e., 4 days total, RSL3^R^ DH and ML210^R^ DH) (Figure 1A).

### 4.4 CRISPR‐Cas9‐Mediated Gpx4 Knockout in 4T1 Cells

Gpx4 knockout cell lines were generated in both parental 4T1 and RSL3‐resistant 4T1 cells using a CRISPR‐Cas9 system delivered via lentiviral transduction. The LCV2_Blast vector encoding a sgRNA targeting mouse Gpx4 (sgRNA#1, sequence: caccGCATGCCCGATATGCTGAGTG) or the empty LCV2_Blast vector (serving as a control) were used, as previously validated [46]. HEK293T cells were co‐transfected with 5 µg of either the LCV2_Blast‐Gpx4 sgRNA or empty vector, 5 µg psPAX2, and 0.5 µg pMD2.G packaging plasmids using Lipofectamine 3000 according to the manufacturer's protocol.

Viral supernatants were collected every 24 h for 48 h post‐transfection and filtered through a 0.45 µm membrane. For infection, 4T1 target cells were exposed to the lentiviral supernatant supplemented with 8 µg/mL Polybrene (Sigma‐Aldrich, H9268). Transduced cells were selected with 5 µg/mL blasticidin and 1 µm Liprox until confluency was reached. Subsequently, single‐cell cloning was performed to isolate individual clones. Both control (empty vector) and *Gpx4* ^−/−^ clones were expanded in media containing blasticidin and Liprox to ensure cell survival during selection. Successful knockout of *Gpx4* was confirmed by Western blot analysis (Figure S3G). All cell lines were routinely maintained in the presence of 1 µm Liprox before the experiments.

### 4.5 Mouse Studies and Xenograft Assays

All mouse experiments complied with the relevant ethical regulations and were performed in accordance with a protocol reviewed and approved by the Institutional Animal Care and Use Committee at the Harvard T.H. Chan School of Public Health (protocol IS00003460). All mouse experiments were designed and conducted following the OBSERVE guidelines for the refinement of rodent cancer models [47]. Balb/c (RRID: IMSR_JAX:000651) and NOD.Cg‐Prkdc^scid^Il2rg^1Wjl^/SzJ (NSG, RRID: IMSR_JAX:005557) mice were purchased from Jackson Laboratory. No formal randomization techniques were used; however, treatment groups and specimens were processed in an arbitrary order. The maximum permitted tumor diameter was 2 cm and this limit was not exceeded in any experiment. For all experiments, mice were kept on normal chow and fed ad libitum.

TNBC cell lines were injected into 6‐ to 8‐week‐old female Balb/c or NSG mice. Orthotopic transplantations were performed by TNBC cell injection in the fourth mammary fat pad of mice. Briefly, TNBC cells were washed in PBS and suspended in PBS at a final concentration of 1 × 10^5^ or 1 × 10^6^ 4T1‐ and M231‐derived cells per 100 µL of PBS per mouse respectively (parental or RSL3^R^ or ML210^R^ cells). Mice were anaesthetized using 2.5% isoflurane and processed for the fourth mammary fat pad injection to transplant orthotopically the TNBC cell lines. Mammary fat pad tumor diameters were measured with calipers (length and width) until any tumor in the mouse cohort reached 1 cm in its length before primary tumor resection or 1.5 cm in its length for the maximal ethical endpoint. At that maximal ethical endpoint, all mice in the cohort were euthanized, per approved protocol, for analysis of primary tumor, and metastatic disease burden. Tumor volume was calculated using the following modified ellipsoid formula [47]:

(1) $$Tumor\;volume\;\left(mm^{3}\right)=\frac{Length\;\left(\mathrm{mm}\right)\;\times \;\;Width\;{\left(\mathrm{mm}\right)}^{2}}{2}$$

Spontaneous metastatic disease was studied by performing survival surgery experiments with the surgical removal of the primary tumor. Briefly, mice bearing 0.8–1 cm (in length) primary tumors received 0.5 mg/kg Buprenorphine‐SR for analgesia. After 10 mins, mice were anesthetized using 3% isoflurane and underwent primary tumor resection using standard sterile surgical procedures. The resected primary tumors were stored at 4°C in media (RPMI for 4T1‐derived cells and DMEM for M231‐derived cells) supplemented with 1% HI‐FBS, 100 IU/mL penicillin, and 100 µg/mL streptomycin until processing. After primary tumor removal and skin suturing (V‐20 needle with 3‐0 silk braided suture, Roboz, Cat#SUT‐1123‐41), mice were maintained at 37°C during anesthesia recovery and monitored for any signs of pain, infection, body weight loss. Post‐surgery mouse activity was monitored for 5 days. Any mice presenting with one or multiple of the listed signs of distress 5‐days post‐surgery were immediately euthanized, per approved protocol, and excluded. Mice's survival was assessed following the scoring criteria form the OBSERVE guidelines and were monitored for clinical signs of illness or distress related to metastatic disease [47]. The pre‐determined endpoint of the survival experiments was day 80 post‐transplantation even if the remaining mice did not exhibit clinical signs of illness or distress and maintained normal behavioral and physical conditions. Metastatic disease burden was evaluated by bioluminescence imaging of visceral organs (see “bioluminescence imaging”) or hematoxylin and eosin (H&E) staining of the lungs (see “H&E lung for lung metastasis quantification”).

TNBC cell dissemination to the lungs from the blood was assessed by direct injection of cancer cells in the bloodstream of 6‐ to 8‐week‐old female Balb/c or NSG mice. Briefly, TNBC cells washed in PBS and suspended in PBS at a final concentration of 1 × 10^4^ cells per 90 µL per mouse. Ten minutes prior to transplantation, cells were treated with 10 µL of vehicle or 10 µm Liprox (final concentration: 1 µm) and incubated at room temperature. Then, intravenous injections were performed by injecting 100 µL per mouse of cell suspension in the tail vein of mice. TNBC cells dissemination to the lungs was evaluated by bioluminescence imaging (see “Bioluminescence Imaging”).

### 4.6 Bioluminescence Imaging

TNBC cell dissemination to the lungs from the blood was assessed by bioluminescence imaging (all TNBC cell lines expressed luciferase). Ten minutes prior to luminescence imaging, mice were injected intraperitoneally with 100 µL of PBS containing 30 mg/mL d‐luciferin monopotassium salt (Revvity Health Sciences, Cat#122799). Mice were imaged using the IVIS Imaging System 200 Series (Caliper Life Sciences). After mouse euthanasia, the visceral organs were surgically removed and imaged ex vivo. The exposure time ranged from 1 to 30 s, depending on the maximum signal intensity, to avoid saturation but exposure time was kept consistent per experiment. To measure background, a non‐tumor‐bearing mouse was imaged. The bioluminescence signal (total photon flux) was quantified with the “region of interest” measurement tools in Living Image software (PerkinElmer, RRID: SCR_014247). TNBC cell dissemination to the lungs was calculated as observed total photon flux across all lung areas minus background total photon flux in negative control mice.

### 4.7 H&E Staining for Lung Metastasis Quantification

Lung tissues were fixed in 10% formalin solution (Sigma‐Aldrich, Cat#HT501128) for 48 h and then transferred to 100% ethanol until processing for histological analysis. Paraffin embedding and H&E staining were performed by the Rodent Histology Department at Harvard Medical School, adhering to their established staining protocols to ensure consistent and high‐quality results. Briefly, lung tissues were first dehydrated in 100% ethanol and xylene before embedded in paraffin solution for 3 h at 60°C. After cooling and paraffin solidification, sections approximately 5 µm thick were cut using a microtome and mounted on silane‐coated glass slides. These slides were then incubated in a thermostat at 65°C for 1 h to ensure proper adhesion of tissue sections to the slides. To prepare the sections for H&E staining, the slides were first dewaxed using xylene and rehydrated through a graded series of ethanol solutions, followed by a final rinse in distilled water. Then, slides were incubated with hematoxylin 2 before bluing for nuclei staining. After rinsing in water, slides were incubated with eosin‐Y and rinsed with water for cytoplasm and extracellular matrix staining. Stained slides were dehydrated through a graded series of ethanol solutions and xylene before mounting. Lung sections were digitalized using an Eclipse Ni‐e microscope (Nikon, Cat#931245) equipped with a 10× objective lens and the NIS‐Elements software (Nikon, RRID: SCR_014329). Areas of tumor regions and lungs were outlined and quantified using ImageJ (RRID: SCR_003070). The percentage of tumor per lung area was calculated as the area of the tumor region divided by the area of the total lung region, multiplied by 100.

### 4.8 Isolation and Ex Vivo Culture of Cancer Cells Derived From Primary Tumors

Resected primary tumors (from 0.8 to 1 cm in length) were dissociated mechanically with a razor blade on ice. Mashed tumors were transferred into 3 mL of pre‐warmed (37°C) digestion media for enzymatic digestion. Briefly, digestion media was prepared fresh and made with RPMI (for 4T1‐derived tumors) or DMEM (for M231‐derived tumors) supplemented with 0.3 U/mL collagenase P (Sigma‐Aldrich, Cat#11249002001), 0.8 mg/mL dispase II (Sigma‐Aldrich, Cat#D4693), 50 U/mL deoxyribonuclease I (Sigma‐Aldrich, Cat#D4527), 1% HI‐FBS, 100 IU/mL penicillin and 100 µg/mL streptomycin. Tumors in digestion media were thoroughly mixed by up‐and‐down agitations and incubated for 10 min at 37°C. Digested cell suspensions were passed through a 70 µm mesh and transferred in ice‐cold collection media made with RPMI (for 4T1‐derived tumors) or DMEM (for M231‐derived tumors) supplemented with 2 mm EDTA, 1% HI‐FBS, 100 IU/mL penicillin, and 100 µg/mL streptomycin. Remaining tumor clumps were subjected to 4 additional cycles of enzymatic digestion (1 h total) or until no clumps were visible. Collected tumor cell suspensions were washed with PBS, and red blood cells were lysed using 1× RBC lysis buffer (CST, Cat#46232S). Tumor cell suspensions were then characterized by flow cytometry or processed for cancer cell isolation.

Cancer cell isolation from tumor cell suspensions was performed using magnetic‐activated cell sorting (MACS) depletion of immune cells to enrich tumor cell suspensions in cancer cells. Briefly, tumor cells were labeled with magnetic beads‐linked anti‐CD45 ((Miltenyi Biotec Cat#130‐052‐301, RRID: AB_2877061) in automacs rinsing solution (Miltenyi Biotec, Cat#130‐091‐222) supplemented with 20% MACS BSA (Miltenyi Biotec, Cat#130‐091‐376) for 15 min at 4°C. Labeled tumor cells were washed with automacs rinsing solution and passed through a magnetic field in MS columns to trap CD45^+^‐immune cells and negatively select the cancer cell‐enriched fraction in the flow through. Isolated cancer cells were then processed for protein analysis by Western blot as well as metabolomic and lipid profiling by mass spectrometry or maintained in culture using regular complete media of 4T1 or M231 cell lines. Culture of tumor‐derived 4T1 and M231 cells was maintained for a maximum of 5 days for ex vivo assessment of GPX4i‐resistance.

### 4.9 Constructs

dsRed2‐P2A‐Luc vector cloned in FUW lentiviral construct was previously described [23]. psPAX2 (RRID: Addgene_12260) and pMD2G (RRID: Addgene_12259) were used as structural and envelope vectors.

### 4.10 Generation of dsRed/Luciferase‐Tagged TNBC Cells by Lentiviral Transfection

4T1 and M231 were transfected with dsRed2 and luciferase for bioluminescence imaging. Briefly, HEK 293T cells were co‐transfected with dsRed2‐P2A‐Luc vector, psPAX2, and pMD2G for lentiviral production (vector mixing ratio of 0.5:0.3:0.2 µg, respectively) using lipofectamine 3000 following manufacturer recommendations (Thermo Scientific, Cat#L3000015). HEK 293T media was changed 5 h after transfection to reduce cell death and replaced with DMEM supplemented with 10% HI‐FBS, 25 µM chloroquine (Sigma‐Aldrich, Cat# C6628), 100 IU/mL penicillin, and 100 µg/mL streptomycin. The lentiviral media supernatant was collected 48 h after transfection, passed through a 0.45‐µm filter, mixed with 8 µg/mL polybrene (Sigma‐Aldrich, Cat#107689), and added to 4T1 and M231 cell lines. Viruses were removed after 24 h, and the infected cells were expanded for 1 week in their respective complete media. dsRed‐positive transfected cells were sorted by flow cytometry through a 70 µm nozzle and analyzed using BD FACS Aria III (RRID: SCR_016695, Flow Cytometry Research Laboratory, Boston Children's Hospital) and DIVA software (RRID: SCR_001456). After sorting, an aliquot of sorted dsRed/luciferase‐tagged TNBC cells was re‐analyzed to check for purity, which was greater than 95%.

### 4.11 Cell Viability Assay

Cell viability assay was performed in 96‐well plates. Parental and GPX4i‐resistant cells derived from 4T1 and M231 cell lines were seeded at 5000 and 10 000 cells in 100 µL of complete RPMI and DMEM, respectively. RSL3^R^ and ML210^R^ cells were treated in duplicate with 1 µm RSL3 and 30 µm ML210, respectively. After 24 h, cells were treated in the presence of the test compounds for 48 h at 37°C, 5% CO_2_. Viability was quantified using the absorbance‐based MTT assay (Sigma‐Aldrich, Cat#M5655). Briefly, MTT was dissolved at 5 mg/mL in PBS using sonication and filtered through a 0.22 µm filter. Cells were incubated with 100 µL of RPMI (for 4T1‐derived cells) or DMEM (for M231‐derived cells) and MTT solution (1:1 ratio) for 3 h at 37°C, 5% CO_2_. Wells with no cells were used as background controls.

MTT‐media solution was removed, and 100 µL DMSO was added to each cell‐containing and background control wells. After 10–60 min incubation at room temperature under agitation, absorbance was read at OD = 590 nm using EnSpire Multimode Plate Reader (PerkinElmer). Viability was determined by calculating the average of the technical replicates and subtracting the background control to obtain the corrected absorbance, with the amount of absorbance being proportional to cell number. Viability was represented relative to the vehicle or untreated conditions multiplied by 100.

### 4.12 Proliferation Assay

Cell proliferation was monitored by live‐imaging. Briefly, parental and GPX4i‐resistant cells derived from 4T1 and M231 cell lines were seeded in 4 technical replicates at 5000 and 10 000 cells in 100 µL of complete RPMI and DMEM, respectively. RSL3^R^ and ML210^R^ cells were treated with 1 µm RSL3 and 30 µm ML210, respectively. Cells were incubated for 5 h at 37°C, 5% CO_2_ prior to live‐imaging to allow cell adherence. The percentage of cell confluency was measured every 4 h by live‐imaging using CellCYTE I (Discover‐Echo) for 72 h total. Cell proliferation and cell death of parental and RSL3^R^ cells derived from 4T1 line stably expressing *Crispr‐Scr* and *Crispr‐Gpx4^−/−^* were monitored by live‐imaging and SYTOX‐Green staining. Briefly, cells were seeded in 4 technical replicates at 5000 cells in 100 µL of complete RPMI with 1 µm Liprox. Cells were incubated for 8 h at 37°C, 5% CO_2_. Then, the cell culture media was removed, and cells were washed once with 1× PBS. 100 µL per well of RPMI without Liprox and with 35 nm SYTOX ‐Green (Invitrogen, Cat#S7020) was added to each well. Percentage of cell confluency and mean fluorescence intensity of SYTOX ‐Green were measured every 4 h by live‐imaging using CellCYTE I (Discover‐Echo) for 72 h total.

### 4.13 BrdU Assay for Cell Cycle Profiling

For in vitro cell cycle profiling, parental and GPX4i‐resistant cells derived from 4T1 and M231 cell lines were seeded in duplicate at 90 000 and 120 000 cells in 1 mL of complete RPMI and DMEM, respectively. RSL3^R^ cells were treated with 1 µm RSL3. Cells were incubated 16 h at 37°C, 5% CO_2_ prior to BrdU assay (BD Biosciences, Cat#559619). Briefly, cells were incubated with complete RPMI (for 4T1‐derived cells) or DMEM (for M231‐derived cells) supplemented with 10 µm BrdU or vehicle for 1 h at 37°C, 5% CO_2_. After BrdU incorporation, cells were trypsinized and washed with ice‐cold PBS. Cells were fixed with BD Cytofix for 30 min and permeabilized with BD Perm/Wash buffer for 10 min following the manufacturer's protocol. DNA was denaturated using 30 µg/10^6^ cells DNase for 1 h at 37°C. Newly synthesized DNA was labeled using FITC‐conjugated antibody against BrdU for 20 min at room temperature. Then, cells were washed, and total DNA was stained using 7‐AAD fluorescent dye. Cell cycle profiles were measured by flow cytometry using LSR Fortessa Analyzer (BD Biosciences) and DIVA software (BD Biosciences, RRID: SCR_001456). Instrument set‐ups including 7‐AAD voltage and compensations were adjusted for adequate separation of cells in apoptosis, G0/1, S phase, and G2+M. A total of 10 000 events were recorded, and the percentage of cells in apoptosis (BrdU‐negative, 7‐AAD‐negative), G0/1 (BrdU‐negative, 7‐AAD‐low), S phase (BrdU‐positive, 7‐AAD‐positive), and G2+M (BrdU‐negative, 7‐AAD‐high) was determined using Flowjo (Becton Dickinson, RRID: SCR_008520).

For in vivo cell cycle profiling, BrdU was administered to tumor‐bearing Balb/c mice. Briefly, mice bearing 1–1.5 cm (in length) primary tumors were treated with 1 mg/kg BrdU or vehicle by intraperitoneal injection (5 mL/kg). After 1 h and 30 min, mice were euthanized, per approved protocol, and primary tumors were resected for mechanical dissociation and enzymatic digestion (see “Isolation and ex vivo culture of cancer cells derived from primary tumors”). After red blood cell lysis, 0.5–1 × 10^6^ tumor cells were labeled with fluorophore‐conjugated antibodies against mouse CD45 (APC, clone 30‐F11, BD, RRID: AB_398672), mouse CD31 (APC, clone MEC 13.3, RRID: AB_398497), mouse Ter‐119/Erythroid cells (APC, clone TER‐119 (RUO), RRID: AB_398635), mouse EpCAM/CD326 (BV421, clone G8.8 (RUO), RRID: AB_2738073) and mouse CD44 (BV786, clone IM7, RRID: AB_2738395) for 20 min at 4°C. All fluorochrome‐conjugated antibodies were purchased from BD Biosciences. Then, cells were washed with PBS and prepared for BrdU assay as described for in vitro cell cycle profiling. Cell cycle profiles (BrdU and 7‐AAD fluorescence signals) were quantified after exclusion of CD45 (immune cells), Ter‐119 (red blood cells), and CD31 (endothelial cells) positive cells in EpCAM+CD44+ cancer cells.

### 4.14 Flow Cytometry Measurement of ROS and Lipid Peroxidation

For in vitro quantification, parental and GPX4i‐resistant cells derived from 4T1 and M231 cell lines were seeded in duplicate at 90 000 and 120 000 cells in 1 mL of complete RPMI and DMEM, respectively. RSL3^R^ and ML210^R^ cells were treated with 1 µm RSL3 and 30 µm ML210, respectively. Cells were incubated 16 h at 37°C, 5% CO_2_. Before staining, cells were trypsinized and washed with PBS. Lipid peroxidation was measured using 5 µm C11‐BODIPY (581/591, Invitrogen, Cat#D3861) diluted in PBS. Mitochondrial/nuclear ROS and cytosolic ROS were labeled using 5 µm CellROX‐Green (485/520, Invitrogen, Cat#C10444) and 5 µm CellROX‐DeepRed (640/665, Invitrogen, Cat#C10422) diluted in PBS, respectively. To avoid cross‐reactivity between the fluorescent probes, staining for lipid ROS, mitochondrial/nuclear ROS, and cytosolic ROS was always performed in independent tubes.

Unstained controls were prepared for each cell line to account for the non‐specific fluorescence. Cells were incubated in the dark for 20 min at 37°C without CO_2_ and washed twice with ice‐cold PBS. Then, dead cells were stained with 0.5 µg/mL DAPI fluorescent DNA stain (Sigma‐Aldrich, Cat# D8417) diluted in PBS. ROS levels were analyzed by flow cytometry using LSR Fortessa Analyzer (BD Biosciences) and DIVA software (BD Biosciences, RRID: SCR_001456). A total of 10 000 events in viable cells (DAPI‐negative cells) were recorded. Data were analyzed using Flowjo (Becton Dickinson, RRID: SCR_008520). To quantify lipid peroxidation levels, we measured the median fluorescence intensity (MedFI) of oxidized BODIPY (FITC‐A) and reduced BODIPY (PE‐E) in viable cells and calculated the lipid peroxidation ratio as previously described following this formula [48]:

(2) $$\begin{array}{ccc} & & Lipid\;peroxidation\;ratio\\ & & \;=\frac{MedFI\;Bodipy_{Ox}\;\;-\;MedFI\;Bodipy_{Ox}\left(Unstained\;samples\right)}{MedFI\;Bodipy_{Red}\;-\;MedFI\;Bodipy_{Red}\left(Unstained\;samples\right)}\;\;\; \end{array}$$

Lipid peroxidation ratios were represented relative to the parental cell lines. To quantify mitochondrial/nuclear ROS and cytosolic ROS levels, we measured the MedFI of CellROX‐Green (FITC‐A) and CellROX‐DeepRed (APC‐A) in viable cells, respectively. Then, the MedFI of the unstained controls was subtracted to the MedFI of the stained cells before representing the ROS levels relative to the parental cell lines.

For in vivo quantification of lipid peroxidation, we used 0.5–1 × 10^6^ cells isolated from primary tumors (see “Isolation and ex vivo culture of cancer cells derived from primary tumors”). After red blood cell lysis, cells were co‐stained with 5 µm C11‐BODIPY (581/591, Invitrogen, Cat#D3861) and fluorophore‐conjugated antibodies against mouse CD45 (APC, clone 30‐F11, BD, RRID: AB_398672), mouse CD31 (APC, clone MEC 13.3, RRID: AB_398497), mouse Ter‐119/Erythroid cells (APC, clone TER‐119 (RUO), RRID: AB_398635), mouse EpCAM/CD326 (BV421, clone G8.8 (RUO), RRID: AB_2738073) and mouse CD44 (BV786, clone IM7, RRID: AB_2738395) for 20 min at 37°C without CO_2_. Then, cells were washed twice with ice‐cold PBS, and dead cells were stained with 5 µL/10^6^ cells 7‐AAD fluorescent DNA stain (Biolegend, Cat#420404) before flow cytometry analysis. To quantify lipid peroxidation levels in cancer cells, we measured the MedFI of oxidized BODIPY (FITC‐A) and reduced BODIPY (PE‐E) after exclusion of DAPI (dead cells), CD45 (immune cells), Ter‐119 (red blood cells), and CD31 (endothelial cells) positive cells in EpCAM+CD44+ cancer cells and calculated the lipid peroxidation ratio. Lipid peroxidation ratios were represented relative to cancer cells derived from parental tumors.

### 4.15 Flow Cytometry Measurement of Immune Infiltration

Tumor immune infiltration was evaluated in 0.8–1 cm in length 4T1‐derived tumors from Balb/c mice in 5 × 10^5^ isolated tumor cells (see “Isolation and ex vivo culture of cancer cells derived from primary tumors”). After red blood cell lysis, cells were stained with fluorophore‐conjugated antibodies for 20 min at 37°C. Cells were washed twice with ice‐cold PBS, and dead cells were stained with either 0.5 µg/mL DAPI (Sigma‐Aldrich, Cat# D8417), 5 µL/10^6^ cells 7‐AAD (Biolegend, Cat#420404), or 0.2 µg/mL PI (Thermo Scientific, Cat#P1304MP) in PBS. Precision Count Beads (Biolegend, 424902) were added to all samples to obtain the absolute counts of each immune subpopulation in the initial 5 × 10^5^ isolated tumor cells. Tumor immune infiltration was analyzed by flow cytometry using LSR Fortessa Analyzer (BD Biosciences) and DIVA software (BD Biosciences, RRID: SCR_001456). Data were analyzed using Flowjo (Becton Dickinson, RRID: SCR_008520). The fluorophore‐conjugated antibodies specific to murine cells used are: B220 (PE/Cyanine7, Biolegend, RRID: AB_313005), CD3 (AF488, Biolegend, RRID: AB_389301), CD4 (PerCP, Biolegend, RRID: AB_893325), CD8a (APC, Miltenyi Biotech, RRID: AB_2728039), CD11c (PerCP, Biolegend, RRID: AB_2129643), CD45 (BV711, Biolegend, RRID: AB_2564383), CD163 (APC, Biolegend, RRID: AB_2814059), F4/80 (PE, BD Biosciences, RRID: AB_2687527), Gr1 (AF647, Biolegend, RRID: AB_1134159), IFN𝛾 (PE, Biolegend, RRID: AB_315402), MHC‐II (BV421, Biolegend, RRID: AB_2650896) and PD‐1 (PE/Cyanine7, Biolegend, RRID: AB_572016).

### 4.16 Western Blot Analysis

Cells were washed twice with ice‐cold PBS and lysed in 1× RIPA buffer (CST, Cat#9806) for 20 min at 4°C under agitation. After full speed centrifugation, protein lysates were collected, and protein concentrations were determined using Pierce BCA Protein Assay Kit (Thermo Scientific, Cat#23227) following the manufacturer's protocol. Protein extracts were supplemented with reducing SDS‐Sample Buffer (Boston BioProducts) and heated for 5 mins at 90°C. Then, proteins were separated using 4% to 15% gradient polyacrylamide SDS‐PAGE gels (Biorad) and electrotransferred to a 0.2 µm nitrocellulose membrane (Biorad). After blocking in Tris‐buffered saline (TBS, Boston BioProducts) with 0.1% Tween‐20 (Sigma‐Aldrich, Cat#P1379) and 5% non‐fat dry powder milk (RPI), membranes were blotted overnight at 4°C with the appropriate primary antibodies. Primary antibodies were detected using the appropriate secondary antibodies against mouse (RRID: AB_3096013) or rabbit (RRID: AB_621843) IgG conjugated to IRDye 800CW. Immunoreactive bands were visualized by fluorescence intensity measurement with the LI‐COR Odyssey CLx Imager using the LI‐COR acquisition software (LICORbio). Protein levels were quantified using the LI‐COR acquisition software and normalized to actin as a non‐variable protein and represented relative to parental cells. Primary antibodies used were: actin (MP Biomedicals, RRID: AB_2335127), ACSL3 (Abcam, RRID: AB_3683571), ACSL4 (Santa Cruz, RRID: AB_10715092), E‐Cadherin (CST, RRID: AB_2291471), FSP1 (Proteintech, RRID: AB_2918791 for 4T1‐derived cells, and Santa Cruz, RRID: AB_2893240 for M231‐derived cells), GCLC (Santa Cruz, RRID: AB_2736837), GPX4 (Abcam, RRID: AB_10973901), Vimentin (CST, RRID: AB_10695459) and xCT (CST, RRID: AB_2800296 for 4T1‐derived cells, and CST, RRID: AB_2687474 for M231‐derived cells).

### 4.17 NADP+/NADPH and GSH/GSSG Measurements

NADP+/NADPH and GSH/GSSG ratios were assessed using NADP/NADPH‐Glo (Cat#G9081) and GSH/GSSG‐Glo (Cat#V6611) assays purchased from Promega. These bioluminescence‐based assays rely on the proportional relationship between the luminescent signal and the amount of NADP+/NADPH and GSH/GSSG, respectively. Cell dilution analyses were performed to determine the optimal cell plating density that are in the linear range of absolute light signal intensity. NADP+/NAPH and GSH/GSSG measurements were performed in parallel to accurately assess the cellular redox balance. For the NADP+/NADPH‐Glo assay, 15 000 and 20 000 cells/well of 4T1‐ and M231‐derived cells were seeded in triplicate in 100 µL/well of complete RPMI and DMEM, respectively. For the GSH/GSSG‐Glo assay, 10 000 cells/well of both 4T1‐ and M231‐derived cells were seeded in triplicate in 100 µL/well of complete RPMI and DMEM, respectively. RSL3^R^ cells were treated with 1 µm RSL3. Wells with no cells were used as background controls. Cells were incubated 16 h at 37°C, 5% CO_2_ before measurements of NADP+, NADPH, GSH, and GSSG following the manufacturer's protocols. Standard curves were generated using purified NADP (Sigma‐Aldrich, Cat# N‐5755), NADPH (Sigma‐ Aldrich, Cat# N‐6705), and GSH (provided in the Promega kit), which were prepared in the same buffers used for the experimental sample analyses. The absolute amounts of NADP+, NADPH, GSH, and GSSG in 4T1‐ and M231‐derived cells were then determined using these standard curves. Luminescence was read at OD = 590 nm using Enspire Multimode plate reader (PerkinElmer). NADP+, NADPH, GSH, and GSSG levels were determined by calculating the average of the technical replicates and subtracting the background control to obtain the corrected absorbance. Data were represented relative to the parental cells.

### 4.18 Mass Spectrometry for Metabolomic Analysis

#### 4.18.1 Metabolite Extraction

Cell metabolite extracts were obtained from frozen pellets of 3 × 10^5^–1 × 10^6^ isolated cancer cells from resected primary tumors (see “Isolation and ex vivo culture of cancer cells derived from primary tumors”). After MACS sorting, cells were washed twice in ice‐cold 0.9% NaCl solution, snap‐frozen in liquid nitrogen, and stored at −80°C until processing. Frozen cell pellets were resuspended with 100 µL per 1.5 × 10^5^ cells of ice‐cold extraction buffer (40% methanol, 40% acetonitrile, 20% water, and 0.5% formic acid). After 10 min incubation on ice, ice‐cold NH_4_HCO_3_ was added for neutralization at 15%, and samples were centrifuged at maximum speed for 15 min at 4°C to eliminate debris. Aliquots from each sample were pooled for LC/MS quality controls, and the metabolite extraction buffer was used as a blank control. Metabolite extracts were stored at −80°C until LC/MS analysis.

#### 4.18.2 LC/MS Analyses of Intracellular Central Metabolites

Metabolite extracts were analyzed using a quadrupole‐orbitrap mass spectrometer coupled with hydrophilic interaction chromatography (HILIC). Chromatographic separation was achieved on an XBridge BEH Amide XP Column (2.5 µm, 2.1 mm × 150 mm) with a guard column (2.5 µm, 2.1 mm × 5 mm) (Waters, Cat#186996724). For the gradient, mobile phase A was water: acetonitrile 95:5, and mobile phase B was water: acetonitrile 20:80, both phases containing 10 mm ammonium acetate and 10 mm ammonium hydroxide. The linear elution gradient was: 0–3 min, 100% B; 3.2–6.2 min, 90% B; 6.5–10.5 min, 80% B; 10.7–13.5 min, 70% B; 13.7–16 min, 45% B; and 16.5–22 min, 100% B, with a flow rate of 0.3 mL/min. The autosampler was at 4°C. The column temperature was at 30°C. The injection volume was 5 µL. Needle wash was applied between samples using acetonitrile: methanol: water at 4: 4: 2 (v: v: v). For mass spectrometry, a Q Exactive HF (Thermo Fisher Scientific) mass spectrometer was used. For MS1 acquisition, the MS scanned from 70 to 1000 m/z with switching polarity at a resolution of 120 000 for all experimental samples. The relevant parameters were: sheath gas, 40; auxiliary gas, 10; sweep gas, 2; spray voltage, 3.5 kV; capillary temperature, 300°C; S‐lens, 45. The resolution was set at 120 000 (at m/z 200). Maximum injection time (max IT) was set at 500 ms, and automatic gain control (AGC) was set at 3 × 10^6^.

#### 4.18.3 Data Processing

MS1 raw data files were converted into mzxML using msconvert and imported to EI‐Maven (Elucidata) for targeted metabolomics. Metabolites were identified based on accurate mass and retention time with an in‐house library. MetaboAnalyst software (v6.0, RRID: SCR_015539) was used to perform unpaired one‐factor statistical analysis. We set a filter for those metabolites that displayed at least a ≥1.5‐ or ≤−1.5‐fold difference in peak intensity between groups and achieved a *p*‐value of <0.05. Data were then interrogated for evidence of metabolic pathway (KEGG database) [49] dysregulation using the enrichment analysis tool of MetaboAnalyst. We set a filter for those metabolic pathways that displayed at least a ≥1.5‐ or ≤0.67‐fold difference in enrichment between groups and achieved a *p*‐value of <0.05.

### 4.19 Mass Spectrometry for Lipidomic Analysis

#### 4.19.1 Lipid Extraction

Cell metabolite extracts were obtained from frozen pellets of 3 × 10^5^–1 × 10^6^ isolated cancer cells from resected primary tumors (see “Isolation and ex vivo culture of cancer cells derived from primary tumors”). After MACS sorting, cells were washed twice in ice‐cold 0.9% NaCl solution, snap‐frozen in liquid nitrogen, and stored at −80°C until processing. Lipid extraction buffer was prepared by mixing butanol and methanol (1:1) with 5 mm ammonium formate and SPLASH Lipidomix Mass Spec Standard (1/20th, Avanti Research, Cat#330707). Frozen cell pellets were resuspended with 100 µL per 1.5 × 10^5^ cells of ice‐cold lipid extraction buffer and incubated for 30 min at 4°C under agitation. Then, samples were centrifuged at maximum speed for 10 min at 4°C to eliminate debris. Aliquots from each sample were pooled for LC/MS quality controls, and lipid extraction buffer was used as a blank control. Lipid extracts were stored at −80°C until LC/MS analysis.

#### 4.19.2 LC/MS Analyses of Intracellular Lipids

Lipid extracts were analyzed using a Dionex Ultimate 3000 RSLC system (Thermo Scientific) coupled with a QExactive mass spectrometer (Thermo Scientific). Chromatographic separation was achieved on an ACQUITY UPLC CSH C18 column (130 Å, 1.7 µm, 2.1 mm × 100 mm) (Waters, Cat#186005303) with an ACQUITY UPLC CSH C18 VanGuard pre‐column (130 Å, 1.7 µm, 2.1 mm × 5 m) (Waters, Cat#186005303) with column temperature at 50°C. For the gradient, mobile phase A consisted of an acetonitrile–water mixture (6:4), and mobile phase B was a 2‐propanol‐acetonitrile mixture (9:1), both phases containing 10 mm ammonium formate and 0.1% formic acid. The linear elution gradient was: 0–3 min, 20% B; 3–7 min, 20%–55% B; 7–15 min, 55%–65% B; 15–21 min, 65%–70% B; 21–24 min, 70%–100% B; and 24–26 min, 100% B, 26–28 min, 100%–20% B, 28–30 min, 20% B, with a flow rate of 0.35 mL/min. The autosampler was at 4°C. The injection volume was 5 µL. Needle wash was applied between samples using a mixture of dichloromethane‐isopropanol‐acetonitrile (1:1:1). ESI‐MS analysis was performed in positive and negative ionization polarities using a combined full mass scan and data‐dependent MS/MS (Top 10) (Full MS/dd‐MS2) approach. The experimental conditions for full scanning were as follows: resolving power, 70 000; AGC target, 1 × 10^6^; and maximum injection time (IT), 100 ms. The scan range of the instrument was set to m/z 100–1200 in both positive and negative ion modes. The experimental conditions for the data‐dependent product ion scanning were as follows: resolving power, 17 500; AGC target, 5 × 10^4^; and maximum IT, 50 ms. The isolation width and stepped normalized collision energy (NCE) were set to 1 m/z, and 10, 20, and 40 eV. The intensity threshold of precursor ions for dd‐MS2 analysis and the dynamic exclusion were set to 1.6 × 10^5^ and 10 s. The ionization conditions in the positive mode were as follows: sheath gas flow rate, 50 arb; auxiliary (AUX) gas flow rate, 15 arb; sweep gas flow rate, 1 arb; ion spray voltage, 3.5 kV; AUX gas heater temperature, 325°C; capillary temperature, 350°C; and S‐lens RF level, 55. The ionization conditions in the negative mode were as follows: sheath gas flow rate, 45 arb; auxiliary (AUX) gas flow rate, 10 arb; sweep gas flow rate, 1 arb; ion spray voltage, 2.5 kV; AUX gas heater temperature, 320°C; capillary temperature, 320°C; and S‐lens RF level, 55.

#### 4.19.3 Data Processing

Thermo Scientific LipidSearch software version 5.0 was used for lipid identification and quantitation. First, the product search mode was used during which lipids were identified based on the exact mass of the precursor ions and the mass spectra resulting from product ion scanning. The precursor and product tolerances were set to 10 and 10 ppm mass windows. The absolute intensity threshold of precursor ions and the relative intensity threshold of product ions were set to 30 000 and 1%. Next, the search results from the individual positive or negative ion files from each sample were aligned within a retention time window (±0.25 min), and then all the data were merged for each annotated lipid with a retention time correction tolerance of 0.5 min. The annotated lipids were then filtered to reduce false positives by only including a total grade of A or B or C for PC and SM; otherwise, only including grade A or B. MetaboAnalyst software (v6.0, RRID: SCR_015539) was used to perform unpaired one‐factor statistical analysis. We set a filter for those lipids that displayed at least a ≥1.5 or ≤1/1.5‐fold difference in peak intensity between groups and achieved a *p‐*value of <0.05.

### 4.20 Spatial Lipidomics

Resected primary tumors from 4T1‐Balb/c mice were snap frozen and stored at –80°C until processing. Frozen primary tumors were sectioned at 12 µm thickness with a microtome (CM 1860, Leica, Wetzlar, Germany) and thaw‐mounted on microscopic glass slides before storing at −80°C in a vacuum until further use. Prior to matrix‐assisted laser desorption/ionization (MALDI) mass spectrometry imaging (MSI) measurements (Spatial Lipidomics), tissue sections were thawed for 20 min in a desiccator at room temperature to avoid any water condensation followed by inspection of the section via optical microscopy (VHX‐5000; Keyence, Osaka, Japan) before matrix application. The matrix 2,5‐dihydroxyacetophenone (DHAP) was deposited via a sublimation chamber on the tissue sections [50]. In short, the apparatus was evacuated to 0.5 mbar, and the cooling finger was filled with an ice–water–NaCl mixture at −15°C. After 4 min, the sublimation chamber was lowered into a heated oil bath at 130°C for 4 min until all matrix crystals have been sublimated. The sample was placed immediately in a desiccator for 5 min prior measurement. Afterward, the matrix layer was inspected via optical microscopy. All MALDI‐MSI experiments were performed using a high‐resolution atmospheric‐pressure autofocusing MALDI imaging ion source (AP‐SMALDI5 AF, TransMIT GmbH, Giessen, Germany) coupled to an orbital trapping mass spectrometer (Thermo Scientific Q Exactive HF, Thermo Fisher Scientific, Bremen, Germany) [51]. Experiments were performed with a 10 µm step size, scanning the range of m/z 400–1200 in positive‐ion mode. The mass resolution was set to 240 000 at m/z 200. Measurements were performed with 50 laser pulses per pixel at a wavelength of 343 nm. Capillary temperature was set to 275°C, the S‐lens was set to 80, and the acceleration voltage was set to +3.5 kV. The mass spectrometer was calibrated externally before measurements using 2,5‐dihydroxy benzoic acid (DHB) clusters [52]. Mass spectrometric images were generated with LipostarMSI (version 2.1.0b7, Molecular Horizon srl, Bettona, Italy) with a bin width of m/z 0.005 and normalized to the total ion current (TIC) and hotspot removal above 99.5th percentile [53]. Assignments of signals based on accurate mass were performed using the LipidMAPS database in LipoStarMSI with mass accuracies required to be 3 ppm or better and cross‐validation by LC‐MS/MS annotation lists (see Mass spectrometry for lipidomic analysis—data analysis). After MALDI‐MSI measurements, H&E stains of the same sample sections were performed and used to determine regions of interest (ROI) containing tumor or non‐tumor tissue. Results were used to define ROIs in LipostarMSI to extract spatial lipidomics data for further analysis of region‐specific lipid profiles.

### 4.21 Bulk RNA Sequencing

RNA extracts were obtained from frozen pellets of 1 × 10^6^ cells. Total RNA was extracted and treated with DNase using the RNeasy Mini Kit (QIAGEN, cat. #74104) according to the manufacturer's instructions. Concentration and quality of RNA were measured with Qubit (Invitrogen, Waltham, MA, USA) and Agilent Bioanalyzer RNA nano/pico chips (Agilent, Santa Clara, CA, USA). Library preparation was performed using Watchmaker RNA Library Prep (Watchmaker Genomics, CO, USA). The samples were sequenced on a Nextseq 2000 P2 XLEAP (100 cycles) (Illumina, San Diego, CA, USA) with 2 × 50 bases. Sequences were trimmed with TrimGalore (v.0.6.0) and aligned with hisat2 to either hg38 or mm10 [54]. Gene set enrichment analysis was performed using GSEA (v4.4.0) with 1000 gene set permutations and hallmarks, GO biological processes and molecular functions, KEGG Legacy, Wiki pathways, and REACTOME gene sets (v2025.1).

### 4.22 Statistical Methods

Statistical analyses were conducted using GraphPad Prism software v10.4.2 (RRID: SCR_002798). To test for normal distribution, we performed the Shapiro–Wilk test. For experiments with 2 groups, statistical significance was determined by calculating *p‐*values using a two‐sided unpaired *t‐*test (when a parametric test was appropriate) or two‐sided Mann–Whitney test (when a non‐parametric test was appropriate). For experiments with more than one group, statistical significance was determined by calculating *p* values using one‐way ANOVA or two‐way ANOVA (when a parametric test was appropriate) followed by Tukey's multiple comparisons or Kruskal–Wallis tests (when a non‐parametric test was appropriate) followed by Dunn's multiple comparisons adjustment. Statistical significance of survival distribution was determined by calculating *p*‐values using the Mantel–Cox log‐rank test. No data were excluded unless indicated. Specifically, outliers were excluded using the ROUT test in the immune infiltration profiling. Also, mice sometimes require euthanasia due to a surgical complication. In these instances, the resected primary tumors were analyzed and included, but the endpoint metastatic burden was not. In general, a minimum of *n* = 3 independent experiments was performed, and sample sizes were increased in more complex experiments to ensure reproducibility. All data represent the mean ± s.d. from at least three independent experiments, except for spatial lipidomic for which two independent murine samples were analyzed per group.

## Ethics Statement

All mouse experiments complied with the relevant ethical regulations and were performed in accordance with a protocol reviewed and approved by the Institutional Animal Care and Use Committee at the Harvard T.H. Chan School of Public Health (protocol IS00003460).

## Conflicts of Interest

The authors declare no conflicts of interest.

## Supporting information




**Supporting File 1**: advs74366‐sup‐0001‐SuppMat.docx.

**Supporting File 2**: advs74366‐sup‐0002‐Sabatier_et_al_SupFigureRevised_V2.1.pdf.

**Supporting File 3**: advs74366‐sup‐0003‐Data.zip.

## Data Availability

The data that support the findings of this study are openly available in FigShare at https://doi.org/10.6084/m9.figshare.31250995, reference number 31250995.
