# Supplementary material for: GPX4 Inhibitor Resistance and Metastatic Features in Triple‐Negative Breast Cancer
Source: Adv Sci (Weinh). 2026 Feb 17;13(23):e23198. doi: 10.1002/advs.202523198 (PMC13104122; doi:10.1002/advs.202523198)
Supplement: Supplementary file 1 — Supporting File 1: advs74366‐sup‐0001‐SuppMat.docx. [file ADVS-13-e23198-s002.docx]

Supporting Information

GPX4 INHIBITOR RESISTANCE AND METASTATIC FEATURES IN TRIPLE NEGATIVE BREAST CANCER

Marie Sabatier, Mayher Kaur, Milena Chaufan, Felix-Levin Hormann, Luiza Martins Nascentes Melo, Mario Palma, Jordan Torpey, Yanshan Liang, Alanis Carmona, Cameron Fraser, Midori Flores, Krystina J. Szylo, Mahsa Yavari, Sheng Hui, Alpaslan Tasdogan, Sven Heiles, Jessalyn M. Ubellacker^*^

**Supplementary Figures Legends**

**Figure S1: GPX4i-resistant cells resist to cell death induced by xCT/GPX4 axis targeting *in vitro*.** (**A**) Parental, ML210^R^ and ML210^R^ DH cells derived from 4T1 cell line were incubated with dose-range RSL3, ML210 and Erastin2 for 48 hours. Cell viability was assessed using an absorbance-based assay (MTT) and results are expressed as a ratio to the untreated condition (n=3 independent experiments). (**B-J**) Parental, RSL3^R^, RSL3^R^ DH, ML210^R^ and ML210^R^ DH cells derived from 4T1 and M231 lines were incubated with the indicated doses of RSL3 (B,C,G), ML210 (D-F) and Erastin2 (H,J) for 48h. Cell viability was assessed using an absorbance-based assay (MTT) and the IC50 were determined by nonlinear regression analyses when more than 50% of cell death was induced (n=3-8 independent experiment). nd^: IC50 unable to be determined as the dose-response curve did not achieve 50% inhibition within the tested dose range; in these conditions, each dot represents the mean of the log-transformed viability value obtained. Data in (**A**) are displayed as mean.

**Figure S2: Viability assessment upon dual inhibition of xCT/GCLC/GPX4 axis and FSP1 *in vitro***. (**A-C**) Parental, RSL3^R^ and RSL3^R^ DH cells derived from 4T1 line were incubated with 3µM ML210, 15 µM viFSP1, 500 nM Erastin2 (E2), 1 mM BSO and 1 µM liproxstratin-1 (Liprox) alone or in combination for 48 hours. Cell viability was assessed using an absorbance-based assay (MTT) and results are expressed as a ratio to the untreated condition (n=3 independent experiments). (**D-E**) Parental, ML210^R^ and ML210^R^ DH cells derived from 4T1 line were incubated with 300 nM RSL3, 15 µM viFSP1, 500 nM E2, 1 mM BSO and 1 µM Liprox alone or in combination for 48 hours. Cell viability was assessed using MTT and results are expressed as a ratio to the untreated condition (n=3 independent experiments). (**G-L**) Parental, RSL3^R^ and RSL3^R^ DH cells derived from M231 line were incubated with 1 µM ML210, 30 µM viFSP1, 500 nM E2, 1 mM BSO, 1 µM Liprox and 10 µM FSEN1 alone or in combination for 48 hours. Cell viability was assessed using MTT and results are expressed as a ratio to the untreated condition (n=3 independent experiments). Data are displayed as mean ± s.d. and were analyzed by one-way ANOVA and Tukey’s multiple comparation.

**Figure S3: Proliferation rate of GPX4i-resistant cells is unchanged *in vitro*.** (**A-B**) Percentage of confluency of parental, RSL3^R^, RSL3^R^ DH, ML210^R^ and ML210R DH cells derived from 4T1 (A) and M231 (B) cell lines measured by live-imaging for 72 hours. (**C-F**) Cell cycle profile evaluated by BrdU incorporation assay in parental, RSL3^R^ and RSL3^R^ DH cell lines. The frequencies of cancer cells in apoptotic, S Phase, G0/G1 and G2+M are shown in (C,E) and the gating strategies are represented in (D,F). (**G**) Immunoblot analysis of protein extracts from 4T1 parental and RSL3^R^ stably expressing *Crispr-Scr* and *Crispr-Gpx4^-/-^* (two independent clones per line labeled as #1 and #2) collected after 24 hours using the indicated antibodies. (**H-I**) Proliferation rate and cell death of parental and RSL3^R^ cells derived from 4T1 line stably expressing *Crispr-Scr* and *Crispr-Gpx4^-/-^* (two independent clones per line labeled as #1 and #2) measured by live-imaging and Sytox-Green staining for 72 hours (n=4-6 independent experiments). Percentage of confluency over time are shown in (H). Representative images of cell death as measured by Sytox-Green at 8-, 24- and 48-hour are shown in (I). Data in (**A,F, H**) are displayed as mean ± s.d. and were analyzed by two-way ANOVA and Tukey’s multiple comparation. Data in (**C,E**) are displayed as mean ± s.d. and were analyzed by one-way ANOVA and Dunnett’s multiple comparation.

**Figure S4: GPX4-inhibitor resistant and parental cells maintain similar redox states *in vitro*.** (**A**) Immunoblot analysis of protein extracts from 4T1 parental, RSL3^R^ and RSL3^R^ DH collected after 24-hour using the indicated antibodies. (**B-C**) Immunoblot analysis of protein extracts from 4T1 parental, ML210^R^ and ML210^R^ DH collected after 24-hour using the indicated antibodies. Protein level quantifications relative to the parental cells are represented in (C) (n=4 independent protein extracts). (**D-E**) Parental, ML210^R^ and ML210^R^ DH cells derived from 4T1 cell line were incubated with 1 µM Liprox or vehicle for 24 hours. Lipid peroxidation was evaluated by C11-BODIPY 581/591 staining. Representative plots (D) and BODIPYox/BODIPYred, ratio of oxidized to reduced BODIPY relative to parental control group (E) are shown (n=5 independent experiments). (**F**) Cytosolic ROS levels measured using CellROX-DeepRed fluorescent probe after 24 hours culture of parental, RSL3^R^, RSL3^R^ DH, ML210^R^ and ML210^R^ DH cells derived from 4T1 and M231 cell lines (n=3-5 independent experiments). (**G**) Mitochondrial/nuclear ROS levels measured using CellROX-Green fluorescent probe after 24 hours culture of parental, RSL3^R^, RSL3^R^ DH, ML210^R^ and ML210^R^ DH cells derived from 4T1 and M231 cell lines (n=3-5 independent experiments). (**H-I**) Relative quantification of GSH (H) and GSSG (I) levels in parental, RSL3^R^, RSL3^R^ DH, ML210^R^ and ML210^R^DH cells derived from 4T1 and M231 cell lines after 24 hours (n=3-4 independent experiments). (**J**) GSH/GSSG ratio in 4T1 ML210^R^ and ML210R^R^ DH relative to parental control group collected after 24-hours (n=3 independent experiments). (**K-L**) Relative quantification of NADP^+^ (K) and NADPH (L) levels in parental, RSL3^R^ and RSL3^R^ DH cells derived from 4T1 and M231 cell lines after 24 hours (n=3 independent experiments). (**M**) NADP^+^/NADPH ratio in 4T1 ML210^R^ and ML210R^R^ DH relative to parental control group collected after 24-hours (n=3 independent experiments). Data in (**C**) are displayed as mean ± s.d. and were analyzed by one-way ANOVA and Dunnett’s multiple comparation. Data in (**E-M**) are displayed as mean ± s.d. and were analyzed by one sample t-test.

**Figure S5: Primary tumors from GPX4i-resistant cells compared to parental cells maintain a similar proliferation rate but form fewer metastases.** (**A-B**) Body weight curves (A) and body weight endpoint (B) of 4T1-Balb/c model (n=10 mice per group) (**C-D**) Body weight curves (C) and body weight endpoint (D) of M231-NSG model (n=7-8 mice per group). (**E-F**) Tumor volume endpoints of 4T1-Balb/c (E) and M231-NSG (F) models (n=7-10 mice per group). (**G**) Experimental schematic of tumor growth assessment and BrdU cell cycle profiling in Balb/c mice transplanted with parental, RSL3^R^ and ML210^R^ cells derived from 4T1 cell line. (**H-I**) Tumor growth curve (H) and tumor volume endpoint (I) of Balb/c mice transplanted with parental, RSL3^R^ and ML210^R^ cells derived from 4T1 cell line (n=5-10 mice per group). (**J**) Cell cycle profile of cancer cells evaluated by BrdU incorporation assay from parental- and RSL3^R^-derived primary tumor in 4T1-Balb/c model. The frequencies of cancer cells in S Phase, G0/G1 and G2+M are represented (n=4 mice per group). (**K-L**) Linear regression analysis of the metastatic areas in lungs and tumor size in 4T1-Balb/c (K) and M231-NSG (L) models (n=6 and n=4 mice per group, respectively). (**M-P**) Cancer cells were isolated from the resected primary tumor in 4T1-Balb/c (M-N) and M231-NSG (O-P) models and incubated with dose-range RSL3 (M,O) and Erastin2 (N,P) for 48 hours. Cell viability was assessed using absorbance-based assay (MTT) and the IC50 were determined by nonlinear regression analyses (n=6-8 mice per group). (**Q**) Immunoblot analysis of protein extracts from cancer cells isolated from the resected primary tumor in M231-NSG using the indicated antibodies. Data in (**A, C**) are displayed as mean ± s.d. and were analyzed by two-way ANOVA and Tukey’s multiple comparation. Data in (**B,D,E,F**) are displayed as mean ± s.d. and were analyzed by two-sided Mann-Whitney test. Data in (**H**) are displayed as mean ± s.d. and were analyzed by Mixed-effect analysis and Dunnett’s multiple comparation (* *P* < 0.05 and ** *P < 0.01*). Data in (**I**) are displayed as mean ± s.d. and were analyzed by Kruskal-Wallis test and Dunn’s multiple comparation. Data in (**J**) are displayed as mean ± s.d. and were analyzed by one-way ANOVA and Šidák’s multiple comparation.

**Figure S6: Characterization of primary tumors from 4T1 GPX4i-resistant cells compared to parental cells in immunodeficient mice.** (**A-B**) Body weight curves (A) and body weight endpoint (B) of D22 cohort in 4T1-NSG model (n=5 mice per group). (**C-D**) Body weight curves (C) and body weight endpoint (D) of Survival cohort in 4T1-NSG model (n=5 mice per group). (**E-F**) Tumor volume endpoints of D22 cohort (E) and Survival cohort (F) in 4T1-NSG model (n=5 mice per group). (**G**) Linear regression analysis of the metastatic areas in lungs and tumor size in 4T1-NSG (n=3 mice per group) (**H-J**) Cancer cells were isolated from the resected primary tumor in 4T1-NSG model and incubated with dose-range RSL3 (H), ML210 (I) and Erastin2 (J) for 48h. Cell viability was assessed using absorbance-based assay (MTT) and the IC50 were determined by nonlinear regression analyses (n=10 mice per group). (**K**) Immunoblot analysis of protein extracts from cancer cells isolated from the resected primary tumor in 4T1-NSG using the indicated antibodies. Data in (**A,C**) are displayed as mean ± s.d. and were analyzed by Mixed-effect analysis and Šidák’s multiple comparation. Data in (**B,D,E,F**) are displayed as mean ± s.d. and were analyzed by two-sided Mann-Whitney test.

**Figure S7: Primary tumors from TNBC GPX4i-resistant cells compared to parental cells display similar immune infiltration profile.** (**A-I**) Immune infiltration profile from parental- and RSL3^R^-derived primary tumors in 4T1-Balb/c model measured by flow cytometry. Absolute cell counts of the indicated immune subpopulation was calculated in 5x10^5^ primary tumor-derived cells for each mouse (n=9-12 mice per group) (**J**) Relative median fluorescence intensity of MHCII in immune cells (CD45-positive) from parental- and RSL3^R^-derived primary tumor in 4T1-Balb/c model measured by flow cytometry (n=11-12 mice per group). (**K,L**) Median fluorescence intensity of IFN𝛾 and PD-1 in CD8a-positive T cells from parental- and RSL3^R^-derived primary tumor in 4T1-Balb/c model measured by flow cytometry (n=9-12 mice per group). All data are displayed as mean ± s.d. and were analyzed by two-sided Mann-Whitney test. Outliers that potentially account for cell count error of the total primary tumor cells analyzed were identified by ROUT test and excluded.

**Figure S8: Metabolic profiles of cancer cells derived from GPX4i-resistant tumor differs between murine models.** (**A-C**) Metabolomic profiling was performed on cancer cells isolated from the resected primary tumor in 4T1-Balb/c (A), 4T1-NSG (B) and M231-NSG (C) by LC/MS (n=5-7 mice per group). Fold change (FC) of metabolite peak intensities and *P*-value for RSL3^R^- versus parental-derived cancer cells comparisons calculated using MetaboAnalyst are shown in and significant changes in metabolite peak intensities are highlighted (FC ≥1.5 and ≤0.66, *P* <0.05).

**Figure S9: Lipidomic profile of primary tumors from 4T1 GPX4i-resistant cells compared to parental cells in Balb/c mice.** (**A-C**) Lipid profiling was performed on cancer cells isolated from the resected primary tumor in 4T1-Balb/c by LC/MS (n=7 mice per group). Lipid peak intensities of significantly increased phospholipids (A), sphingomyelins and ceramides (B) and acylcarnitine and coenzyme Q9 (C) in RSL3^R^- versus parental-derived cancer cells calculated using MetaboAnalyst are shown in (A) (FC ≥1.5, *P* < 0.05). (**D**) Relative cancer cell size measured by flow cytometry from the resected primary tumors of 4T1-Balb/c model (n=7-9 mice per group). (**E-L**) Fatty acyl chain composition of PC (E), PE (F), PE-P (G), PE-O (H), PI (I), PS (J), TG (K) and TG-O (L). Data in (**C,D**) are represented as mean ± s.d. Data in (**D**) were analyzed by two-sided Mann-Whitney test. PC(-O): phosphatidylcholine (-alkyl ether), PE(-P/-O): phosphatidylethanolamine (-plasmalogen/-alkyl ether), PI: phosphatidylinositol, PS: phosphatidylserine, SM: sphingomyelin, Cer: ceramide, AcCa: acylcarnitines, Co(Q9): coenzyme Q9, TG(-O): triglycerides (-alkyl ether).

**Figure S10: Lipidomic profile of primary tumors from 4T1 GPX4i-resistant cells compared to parental cells in NSG mice.** (**A**) Lipid profiling was performed on cancer cells isolated from the resected primary tumor in 4T1-NSG by LC/MS (n=7 mice per group). FC of lipid peak intensities and *P*-value for RSL3^R^- versus parental-derived cancer cells comparisons calculated using MetaboAnalyst are shown and significant changes in lipid peak intensities are highlighted (FC ≥1.5 and ≤0.66, *P* <0.05). (**B**) Relative cancer cell size measured by flow cytometry from the resected primary tumors of 4T1-NSG model (n=10 mice per group). (**C-K**) Fatty acyl chain composition of PC (C), PE (D), PE-P (E), PE-O (F), PI (G), PS (H), TG (I) and TG-O (J). Data in (**B**) are represented as mean ± s.d. and analyzed by two-sided Mann-Whitney test. ChE: cholesterol ester, PC(-P/-O): phosphatidylcholine (-plasmalogen/-alkyl ether), PE(-P/-O): phosphatidylethanolamine (-plasmalogen/-alkyl ether), PI: phosphatidylinositol, PS: phosphatidylserine, SM: sphingomyelin, Cer: ceramide, TG(-O): triglycerides (-alkyl ether).

**Figure S11: Spatial lipid profiling of primary tumors from 4T1 GPX4i-resistant cells compared to parental cells in Balb/c mice.** (**A**) Images of primary tumor sections stained with hematoxylin and eosin (H&E) from resected primary tumor from 4T1-Balb/c model corresponding to the section used for spatial lipidomic profiling showing the tumor and non-tumor area outlines used for ROI quantification. (**B-G**) FC of lipid peak intensities in RSL3^R^ versus parental tumors and non-tumor areas per unsaturation levels in percent of total PCs (B), PC-Os (C), PEs (D), PE-O/-Ps (E), PSs (F) and TG (G). Data in (**B-G**) are represented as median.

**Figure S12: Lipidomic profile of primary tumors from M231 GPX4i-resistant cells compared to parental cells in NSG mice.** (**A-E**) Lipid profiling was performed on cancer cells isolated from the resected primary tumor in M231-NSG by LC/MS (n=5-7 mice per group). Lipid peak intensities of significantly increased phospholipids (A), sphingomyelins and ceramides (B) and acylcarnitine (C) and significantly decreased triglycerides (D-E) in RSL3^R^- versus parental-derived cancer cells calculated using MetaboAnalyst are shown in (A) (FC ≥1.5, *P* < 0.05). (**F**) Relative cancer cell size measured by flow cytometry from the resected primary tumors of M231-NSG model (n=10 mice per group). (**H-N**) Fatty acyl chain composition of PC (H), PE (I), PE-P (J), PE-O (K), PI (L), PS (M), and TG-O (N). Data in (**F**) are displayed as mean ± s.d. and were analyzed by two-sided Mann-Whitney test. AcCa: acylcarnitines, PC(-P/-O): phosphatidylcholine (-plasmalogen/-alkyl ether), PE(-P/-O): phosphatidylethanolamine (-plasmalogen/-alkyl ether), PI: phosphatidylinositol, PS: phosphatidylserine, SM: sphingomyelin, Cer: ceramide, TG(-O): triglycerides (-alkyl ether).

**Figure S13: GPX4i-resistant tumors showed reduced mesenchymal and increased epithelial phenotypes compared to parental tumors.** (**A-C**) bulk RNA sequencing was performed on parental, RSL3^R^, RSL3^R^DH, ML210^R^ and ML210^R^DH lines derived from 4T1 and M231 cells and gene set enrichment analysis using Gene ontology, Hallmarks, REACTOME, Wikipathways and KEGG Legacy 25 databases. Common enriched gene signatures in RSL3^R^ versus parental and RSL3^R^DH versus parental for 4T1 (A) and M231 (B) lines are highlighted. Common enriched gene signatures in ML210^R^ versus parental and ML210^R^DH versus parental for 4T1 are highlighted in (C). (**D-G**) Immunoblot analyses of protein extracts from 4T1 (D) and M231 (E) parental, RSL3^R^ and RSL3^R^DH and 4T1 parental, ML210^R^ and ML210^R^DH (F) collected after 24 hours. Protein level quantifications relative to the parental cells are represented in (G) (n=4 independent protein extracts). (**H-I**) Immunoblot analyses of protein extracts from cancer cells isolated from the resected primary tumor in 4T1-NSG (H) and M231-NSG (I) using the indicated antibodies. Protein level quantification relative to the average of parental control group are represented in (J) (n=6-11 mice per group). (**K**) Relative median fluorescence intensity of EpCAM from parental- and RSL3^R^-derived primary tumor in 4T1-Balb/c, M231-NSG and 4T1-NSG models measured by flow cytometry (n=4-10 mice per group). Data in (**G,J-K**) are displayed as mean ± s.d. and were analyzed by two-sided Mann-Whitney test. E-Cad: E-cadherin.
