# Supplementary figures and images for: GPX4 Inhibitor Resistance and Metastatic Features in Triple‐Negative Breast Cancer
Source: Adv Sci (Weinh). 2026 Feb 17;13(23):e23198. doi: 10.1002/advs.202523198 (PMC13104122; doi:10.1002/advs.202523198)

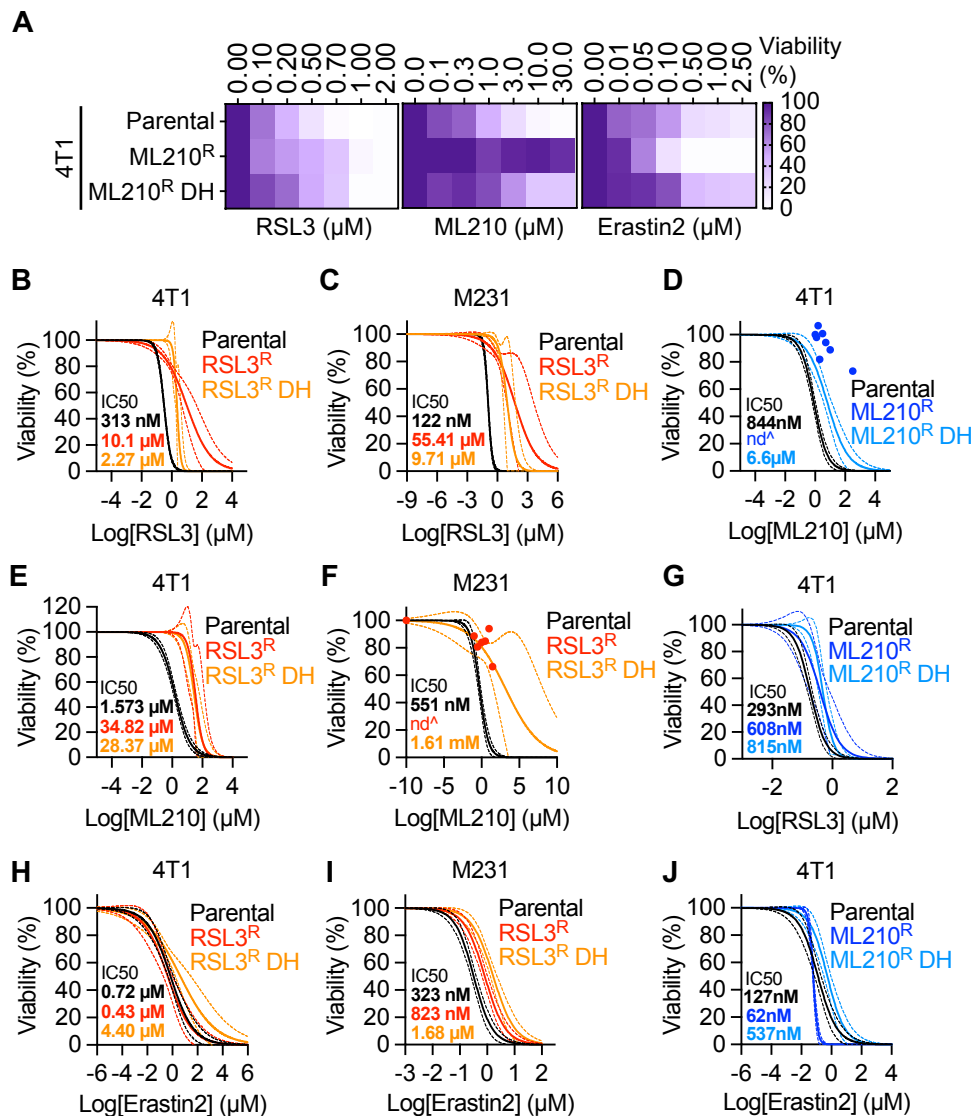

fig. S1

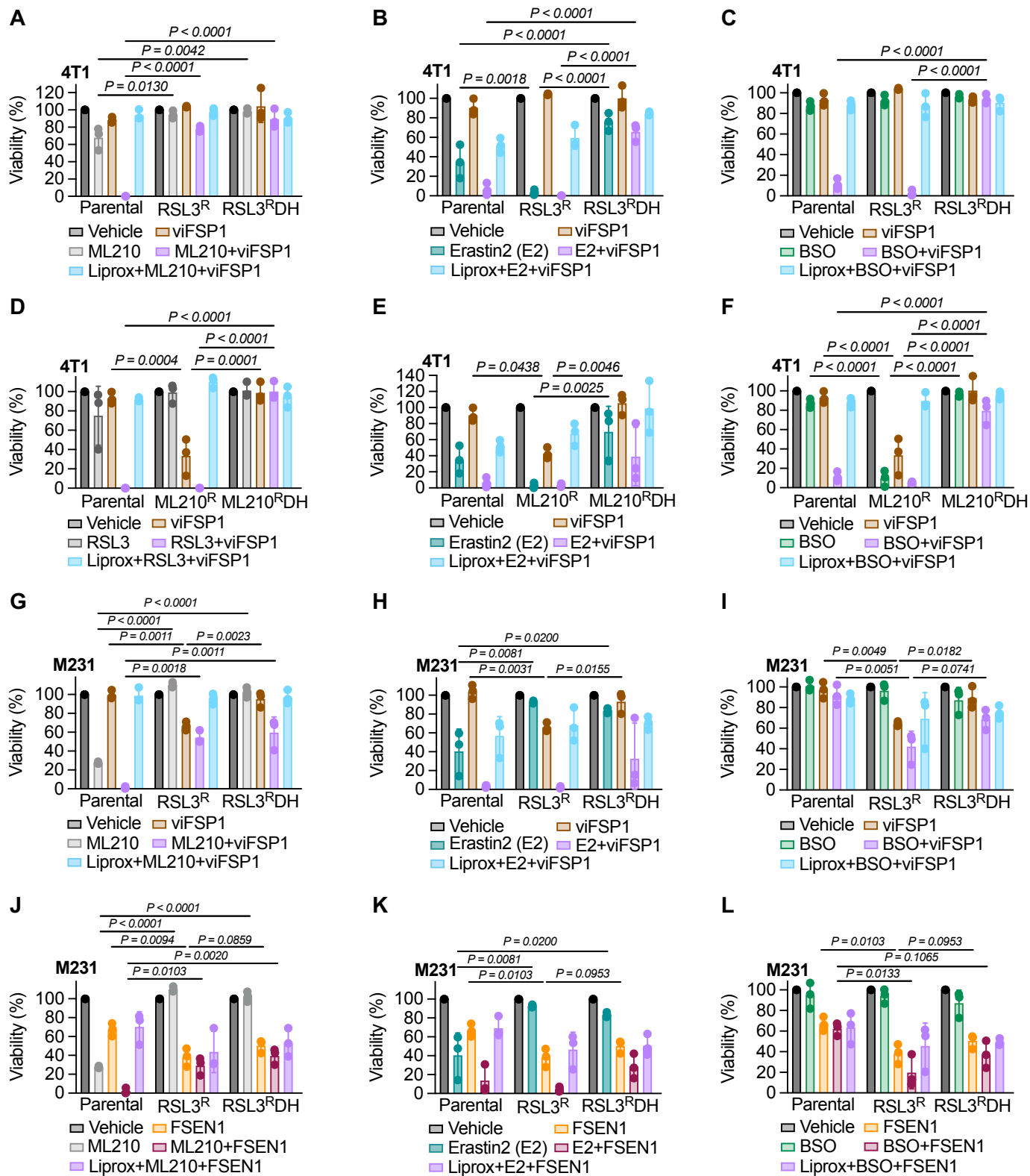

fig. S2

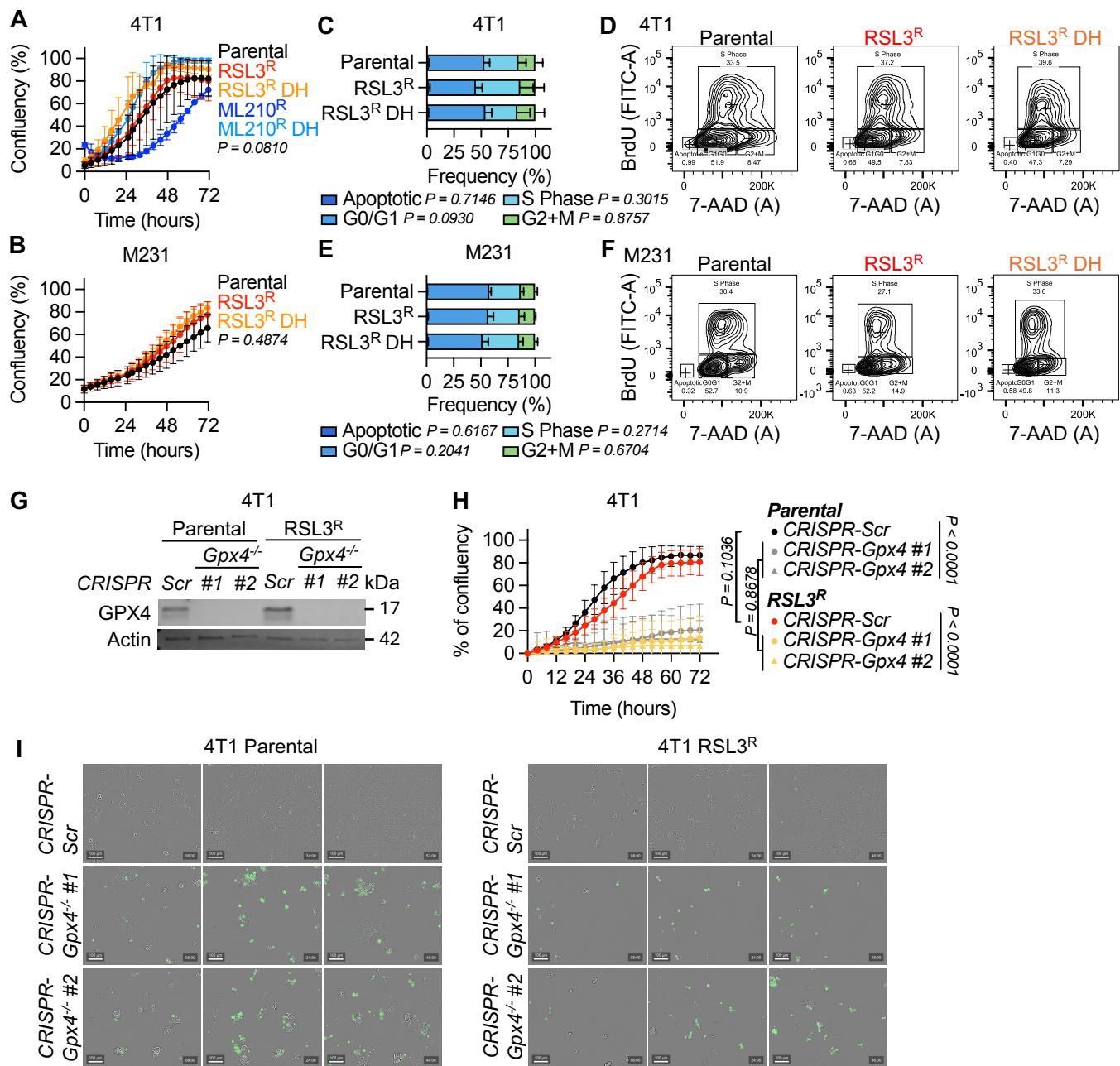

fig. S3

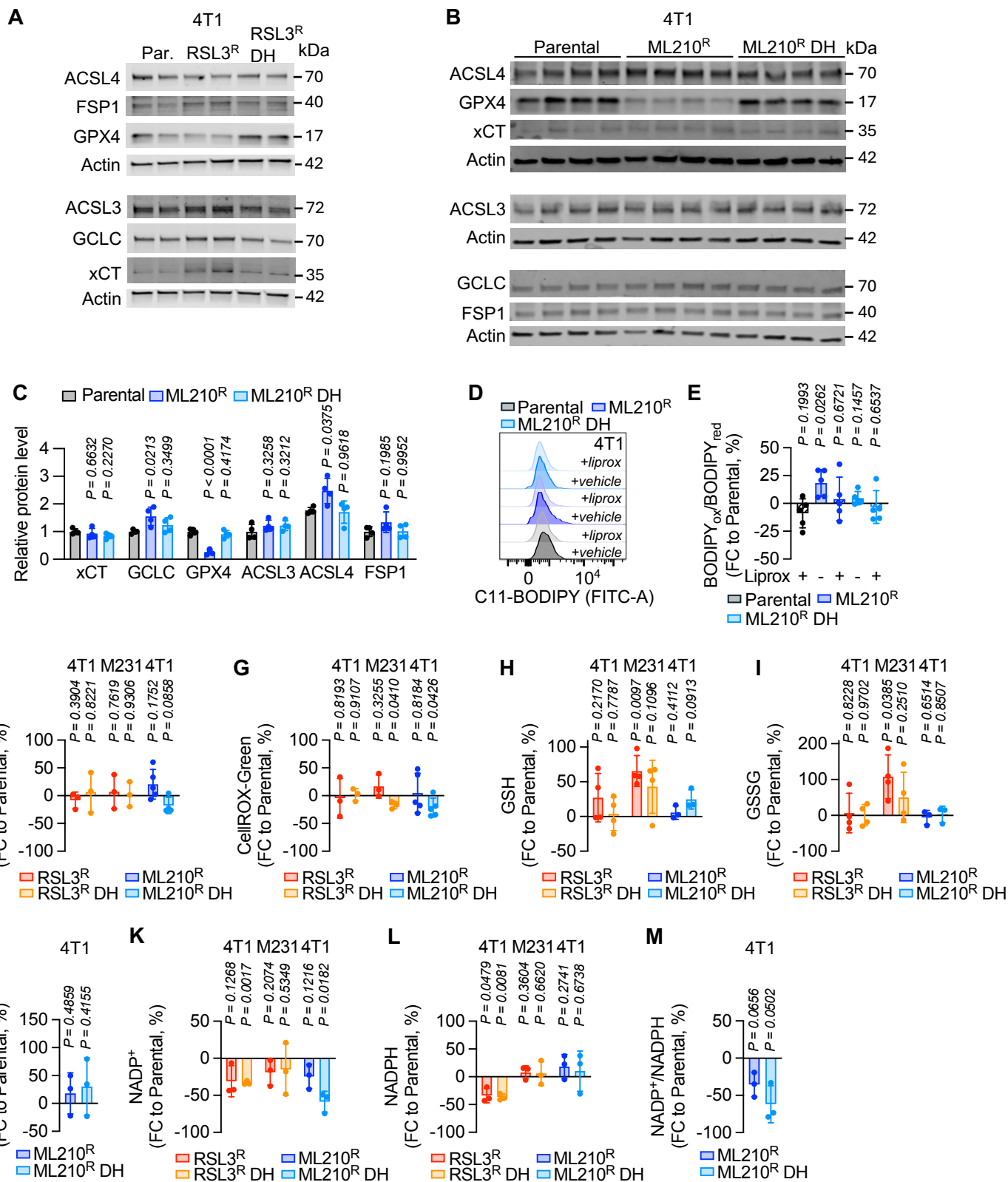

fig. S4

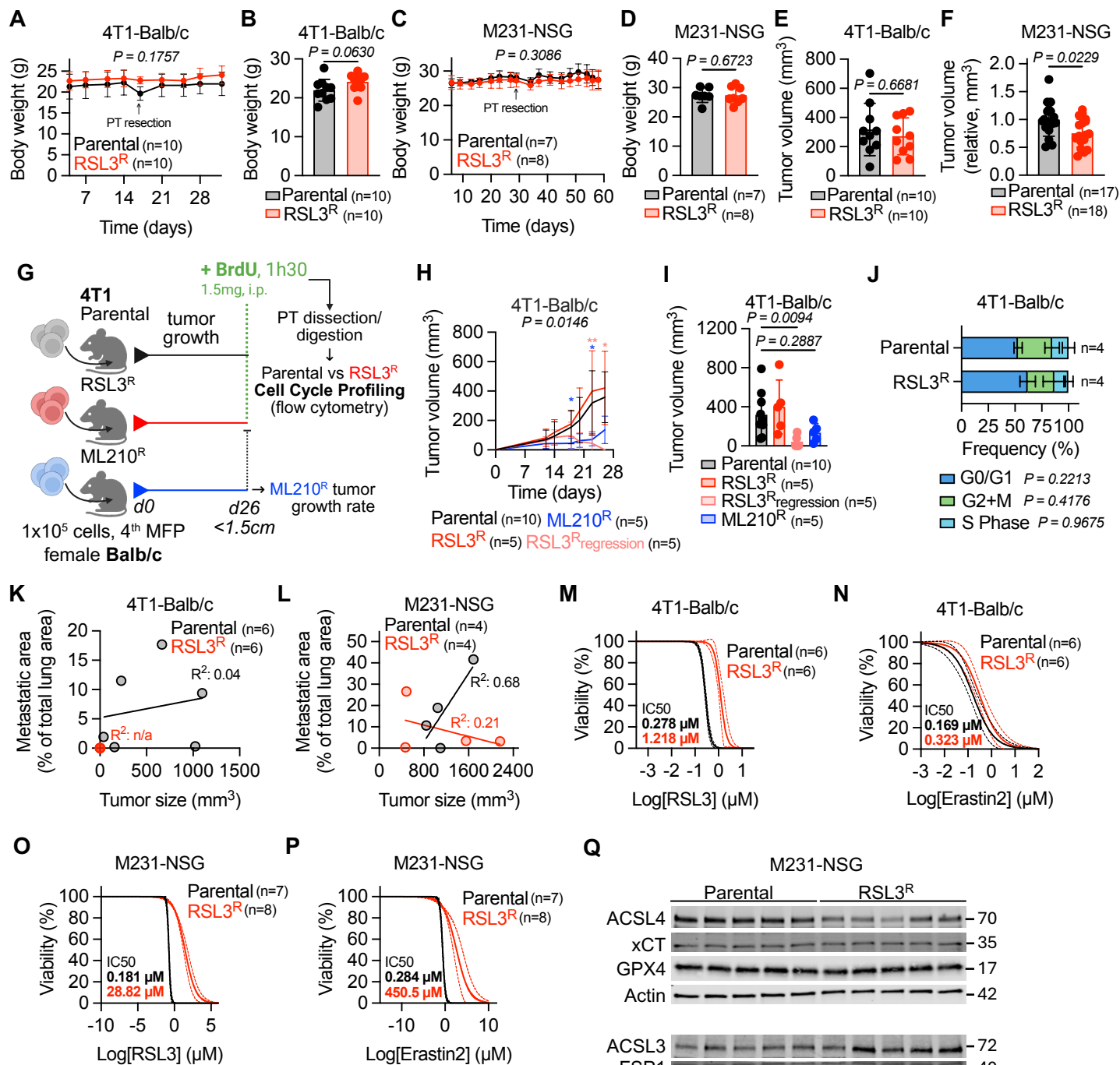

fig. S5

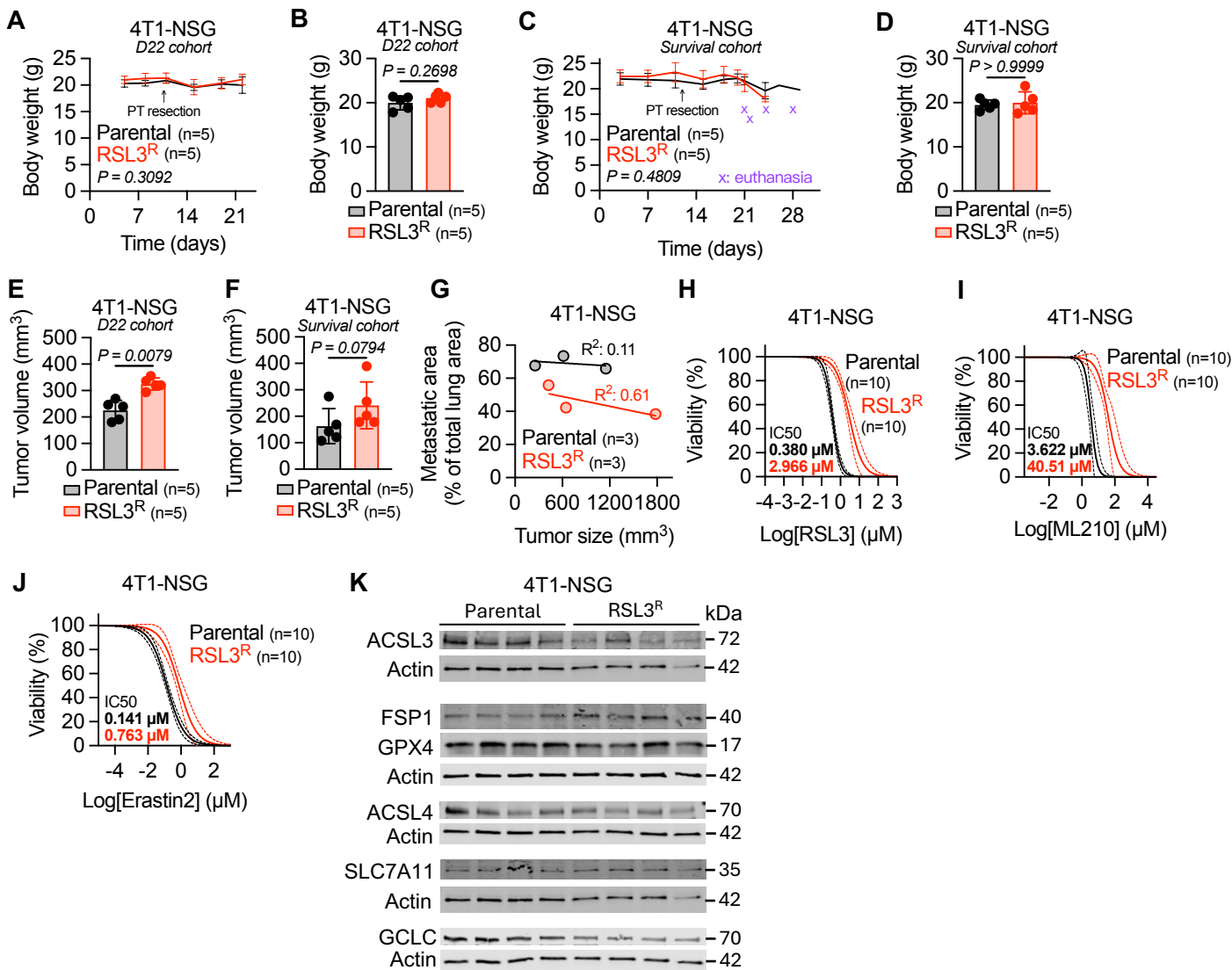

fig. S6

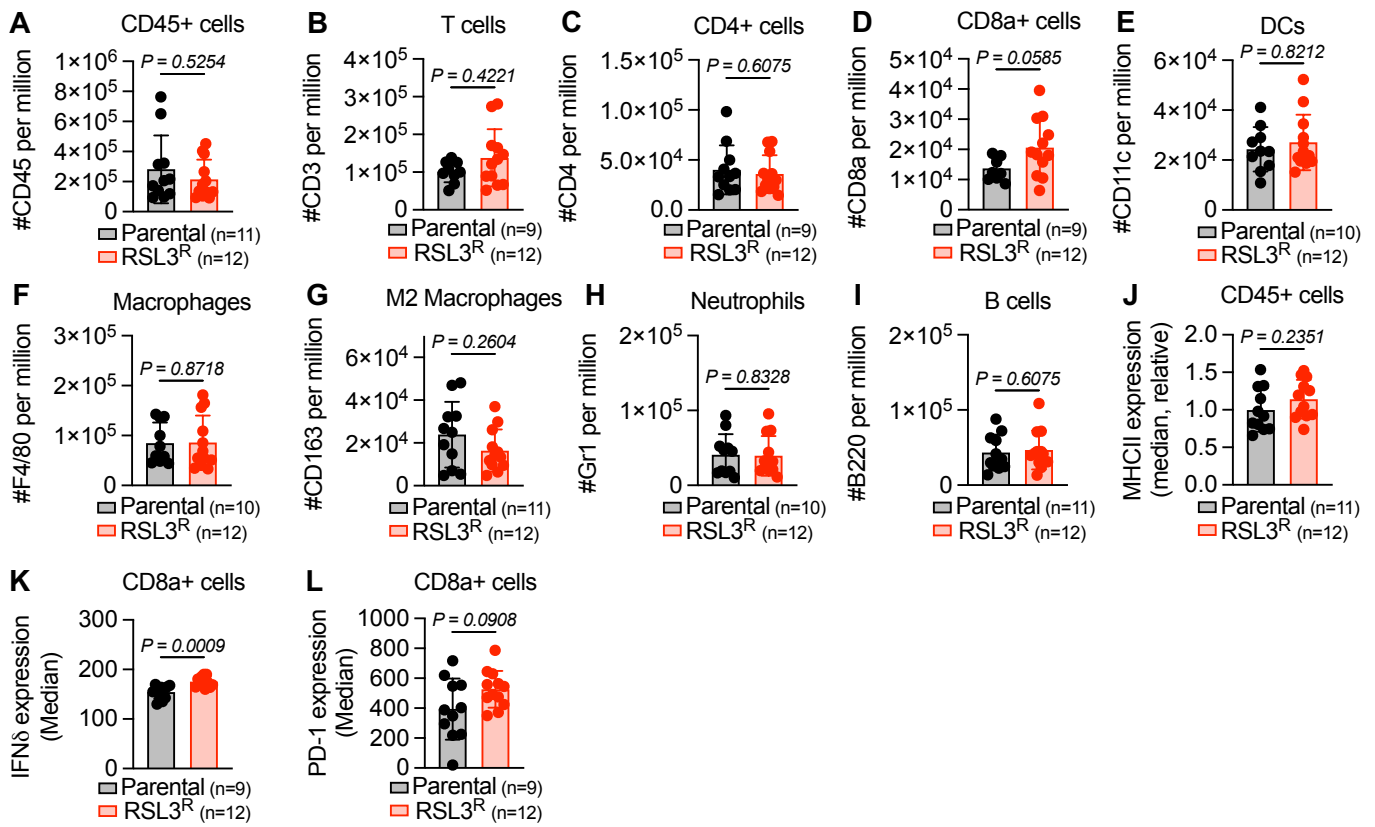

fig. S7

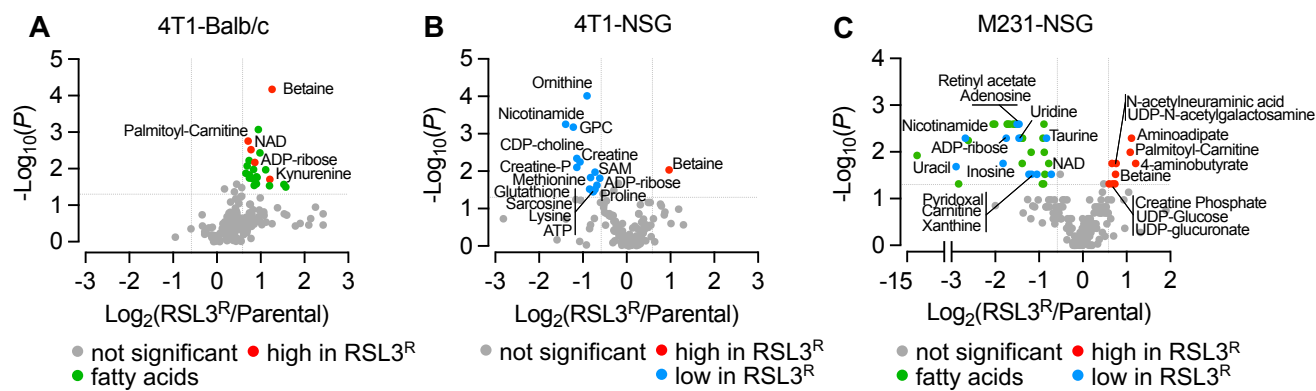

fig. S8

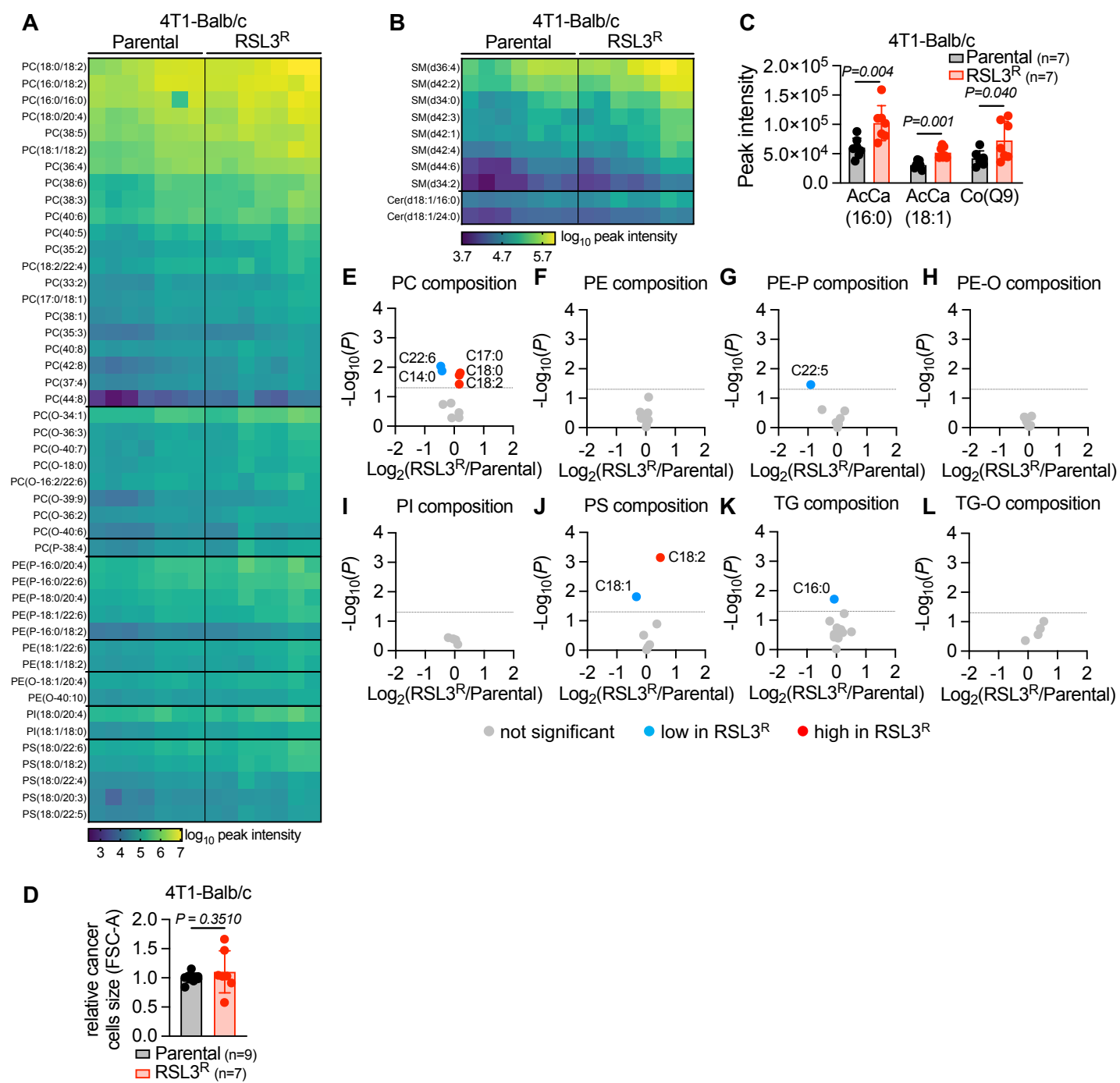

fig. S9

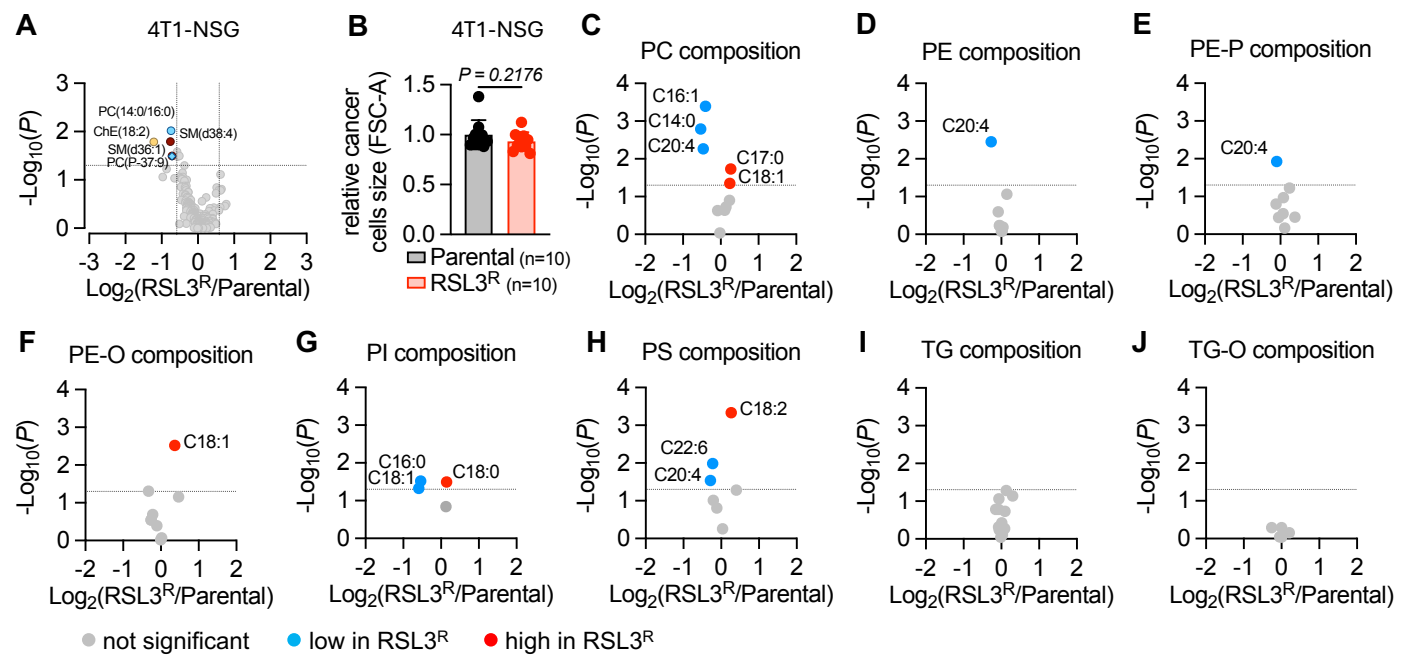

fig. S10

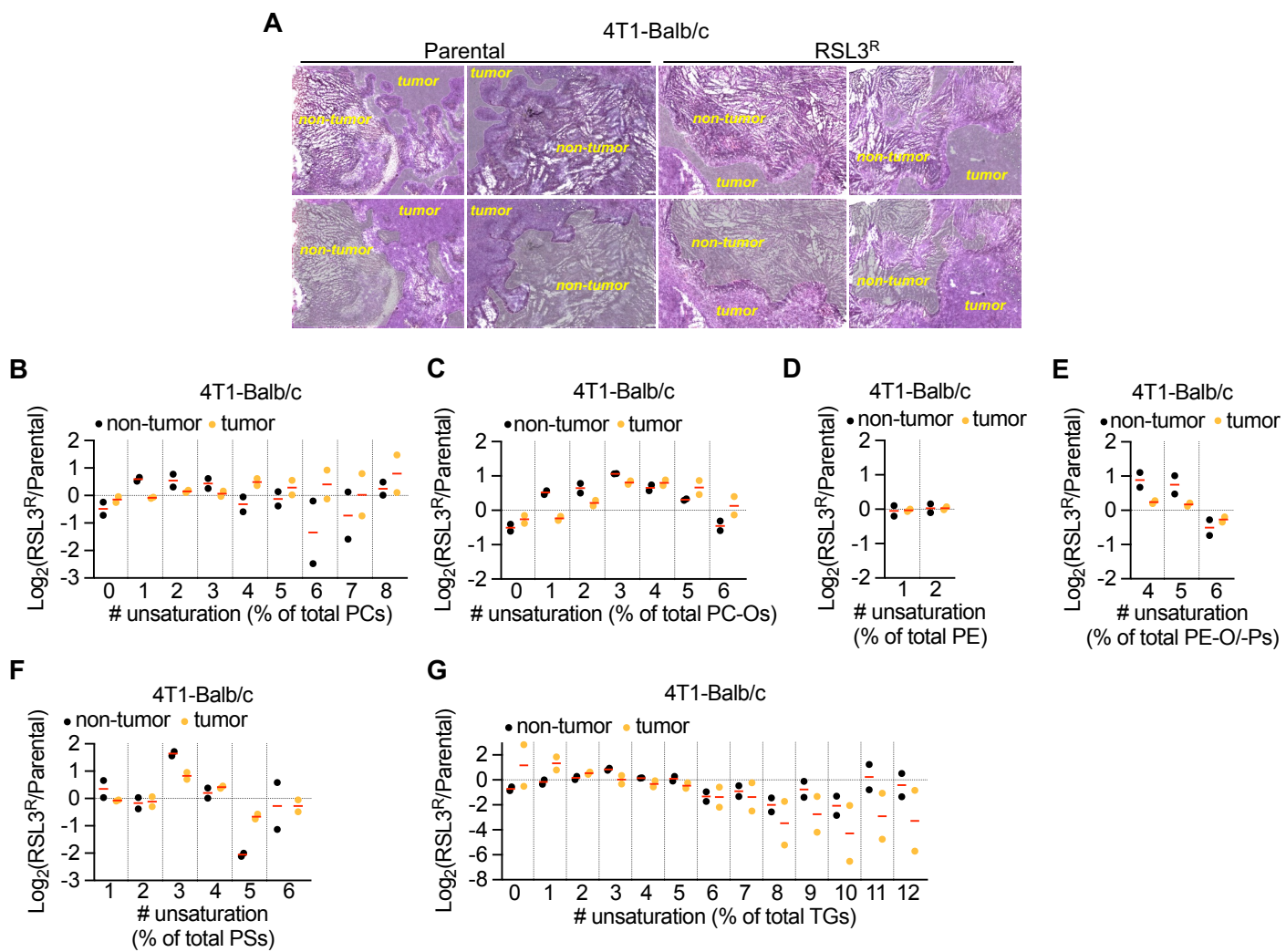

fig. S11

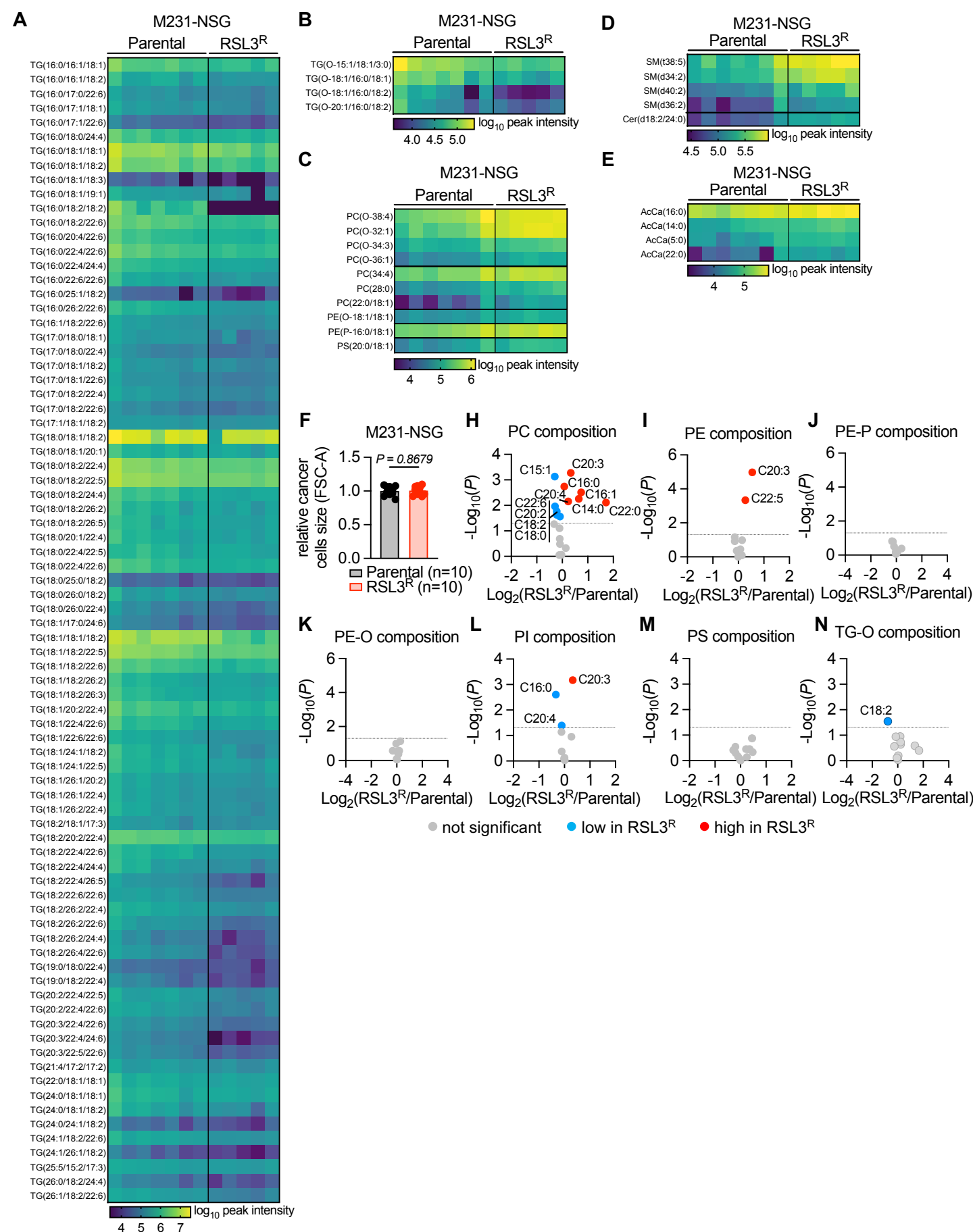

fig. S12

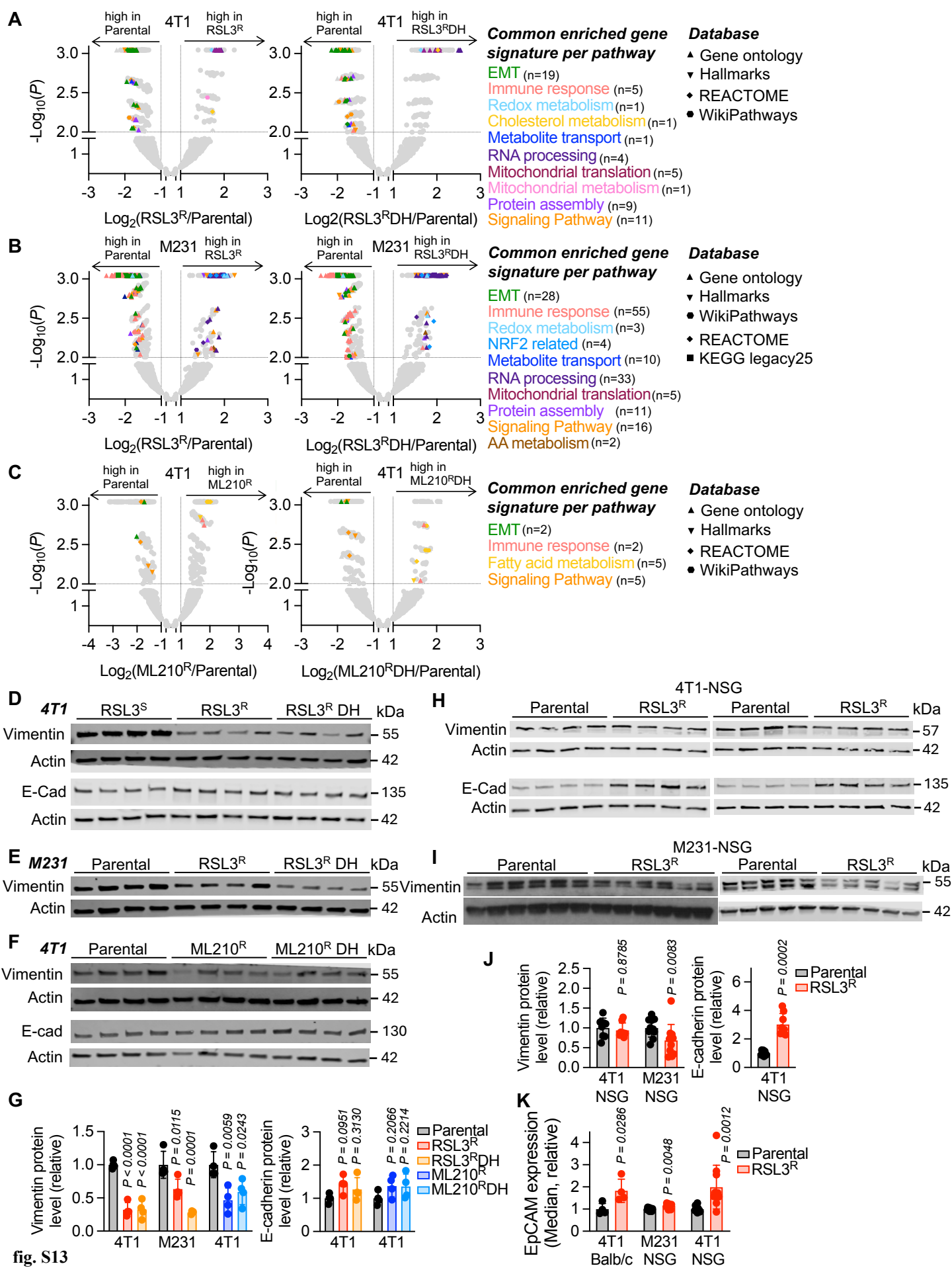

fig. S13

Supplement: Supplementary file 2 — Supporting File 2: advs74366‐sup‐0002‐Sabatier_et_al_SupFigureRevised_V2.1.pdf. [file ADVS-13-e23198-s001.pdf]
